# ﻿*Heterocypris exodonta* sp. nov. (Ostracoda, Cyprididae), morphological and molecular description of a high altitude asexual microcrustacean from the Nam Co region, Southern Tibetan Plateau

**DOI:** 10.3897/zookeys.1264.140174

**Published:** 2025-12-19

**Authors:** Mauricio Bonilla-Flores, Ivana Karanovic, Paula Echeverría-Galindo, Peter Frenzel, Liseth Pérez, Nicole Börner, Katharina Dulias, Junbo Wang, Antje Schwalb

**Affiliations:** 1 Institute of Geosystems and Bioindication, Technische Universität Braunschweig, 38106 Braunschweig, Germany Technische Universität Braunschweig Braunschweig Germany; 2 Department of Life Science, Research Institute for Convergence of Basic Science, Hanyang University, 04763 Seoul, Republic of Korea Hanyang University Seoul Republic of Korea; 3 Institute of Organic Biogeochemistry in Geo-Systems (OBG), RWTH Aachen University, 52056 Aachen, Germany RWTH Aachen University Aachen Germany; 4 Institute of Geosciences, University of Jena, 07749 Jena, Germany University of Jena Jena Germany; 5 Institute of Geosciences, Kiel University, 24118 Kiel, Germany Kiel University Kiel Germany; 6 Max-Planck-Institut für Biogeochemie, 07745 Jena, Germany Max-Planck-Institut für Biogeochemie Jena Germany; 7 State Key Laboratory of Tibetan Plateau Earth System, Environment and Resources (TPESER), Institute of Tibetan Plateau Research, Chinese Academy of Sciences, Beijing 100101, China Institute of Tibetan Plateau Research, Chinese Academy of Sciences Beijing China

**Keywords:** Asexual reproduction, freshwater ecosystems, high altitude, ostracods, taxonomy, temporary ponds

## Abstract

A new ostracod species, *Heterocypris exodonta* Bonilla-Flores & Karanovic, **sp. nov.** is described from a high-altitude temporary pond near Nam Co, Southern Tibetan Plateau. Detailed morphological analyses of valves, soft body, and the partial mitochondrial COI (cytochrome c oxidase subunit I) sequence distinguish this species from its close congeners, *Heterocypris incongruens* and *Heterocypris salina*. Key taxonomic features include the morphology of the female genital lobe and the structure of the internal opening of normal pores, particularly variations in the female genital aperture at the intersection of the seminal duct and the oviduct. Pore openings in *H. exodonta* Bonilla-Flores & Karanovic, **sp. nov.** and *H. incongruens* exhibit a turbine shape, while in *H. salina* they display a simple aperture. Phylogenetic analysis based on the COI dataset supports the new species description and suggests potential synonymy and cryptic species within the *H. salina* and *H. incongruens* complexes. Ecologically, these species mainly inhabit temporary water bodies with high oxygen levels and likely follow an r-type ecological strategy, being opportunistic in colonizing new habitats, reproducing via parthenogenesis, and undergoing rapid population growth. This study enhances the understanding of Southern Tibetan Plateau ostracod biodiversity, providing crucial morphological and molecular data for species identification, particularly for parthenogenetic species lacking standard male morphological characters. Additionally, this research offers methods to prevent misidentifications and misinterpretations in (paleo-)ecological studies, contributing significantly to the broader knowledge of taxonomy and ecology of ostracods from the Southern Tibetan Plateau. Due to the high morphological plasticity of *Heterocypris incongruens*, our findings highlight the need for caution when identifying similar species, as external resemblance may conceal genetic divergence.

## ﻿Introduction

During the past two decades, the documented diversity of identified ostracods from the Southern Tibetan Plateau (STP) has increased from 30 to 89 species (Wrozyna et al. 2009a, b; Yu et al. 2009; Mischke et al. 2010; Mischke 2012; Peng et al. 2021). However, ongoing discoveries highlight the need to address taxonomic inconsistencies within this extensive and largely unexplored region (Mischke 2012; Peng et al. 2021). Genera such as *Heterocypris* Claus, 1892, *Ilyocypris* Brady & Norman, 1889, and the family Candonidae Kaufmann, 1900, present taxonomic challenges, as morphological features are similar and descriptions are mostly available for valves only (Mischke 2012). This has led to potential inaccuracies in estimating biodiversity, as certain cryptic species require a review of soft part morphology and DNA sequences for species differentiation (Echeverría-Galindo et al. 2021).

Nam Co, situated at 4730 m a.s.l. in the south-central part of the STP, is surrounded by temporary ponds that sustain aquatic species adapted to extreme conditions (Blaustein and Schwartz 2001; Jacobsen and Dangles 2017). The genus *Heterocypris*, mainly composed of desiccation-tolerant species, commonly inhabits these temporary habitats (Meisch 2000; Schwalb et al. 2002; Aguilar-Alberola and Mesquita-Joanes 2011; Mischke 2012; Zhai and Zhao 2014; Akita et al. 2016). Despite its high diversity, with 70 known species (Meisch et al. 2024), the genus diagnosis still poses problems in proper species classification. For instance, the right valve has a row of denticles on its anterior and posterior edges, and it is overlapped by the opposing left valve (Purper and Würdig-Maciel 1974; Martens et al. 2019). The same characteristics are present in *Cyprinotus* species, members of the same subfamily, except for the presence of a hump in *Cyprinotus* (Martens et al. 2019; Savatenalinton 2020). The challenge also lies in species delineation due to the remarkably similar soft body morphology (Meisch 2000), highlighting the importance of combining valve characteristics and exploring additional features to differentiate between species.

The presence of cryptic species, morphologically indistinguishable but genetically divergent, further complicates taxonomic differentiation (Bode et al. 2010), impacting ecological and paleoenvironmental assessments (Lord et al. 2012). Resolving taxonomic issues in *Heterocypris*, therefore, requires an integrative taxonomic approach (Padial et al. 2010), combining morphological and molecular methods. Parthenogenetically reproducing ostracods pose additional problems in species delineation. Characters such as the morphology of the female genital opening (often labeled as a lobe) are rarely used for species identification, but they can be useful taxonomic characters (Smith and Kamiya 2007; Bonilla-Flores et al. 2024. These lobes, in external view, are located in the posterior region of the body, near the base of the uropodal ramus. The anterior part has a rigid oval shape, while the posterior part displays a tubular junction (intersection) that internally connects to the oviducts, and also to the seminal ducts (Smith and Kamiya 2007). The appearance of this intersection is variable in the Cyprididae family, such as *Amphicypris argentinensis* (Fontana & Ballent, 2005), *Dolerocypria mukaishimensis* Okubo, 1980 (Karanovic & Lee, 2012), *Chrissia dongqianhuensis* Kong, Karanovic & Yu, 2014 and *Stenocypris major* (Baird, 1859), and *Cypris pretusi* Mesquita-Joanes, Aguilar-Alberola, Palero & Rueda, 2020.

Three *Heterocypris* species have been documented from the STP and the Qinghai province: *H. incongruens* (Ramdohr, 1808), *H. salina* (Brady, 1868), and *H. vandouwei* (Brehm, 1923). While the first two were recorded from both areas, the latter was only found in Qinghai province (Peng et al. 2021). Our study describes a new species of *Heterocypris* discovered in a temporary pond near the Nam Co Research Station (**NAMORS**) in September 2019, thus contributing to the diversity of ostracods on the STP.

The aims of this study are to describe a new species of *Heterocypris*; to identify reliable taxonomic characters that distinguish it from the close congeners, *H. incongruens* and *H. salina*; and to use the mitochondrial COI gene to reconstruct its phylogenetic relationship with selected *Heterocypris* and *Cyprinotus* species.

**Figure 1.**
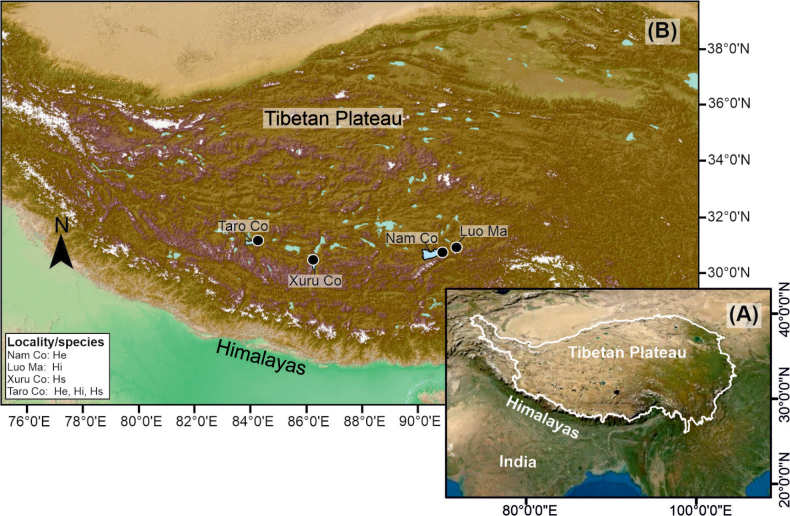
A. Map showing the Tibetan Plateau and B. Sampling localities of *Heterocypris* species within the study area (source: Demis 2023). Abbreviations: He = *Heterocypris exodonta* Bonilla-Flores & Karanovic, sp. nov., Hi = *Heterocypris incongruens* (Ramdohr, 1808), Hs = *Heterocypris salina* (Brady, 1868).

The study focused on sites in the south-central Tibetan Plateau (**STP**), particularly around the lakes Nam Co, Taro Co, Xuru Co, and Luo Ma (Fig. 1). Many of the temporary ponds surveyed are located at elevations higher than 4500 m a.s.l.; however, additional sites at lower altitudes from Mexico and Germany were also included (see Table 1).

**Table 1.** Environmental data from sampling sites of *Heterocypris* species (He = *Heterocypris exodonta* Bonilla-Flores & Karanovic, sp. nov., Hi = *Heterocypris incongruens* (Ramdohr, 1808), Hs = *Heterocypris salina* (Brady, 1868)). WD: water depth, EC: electrical conductivity, WT: water temperature, DO: dissolved oxygen, Alk: alkalinity, ND: no data. (GPS coordinates referenced to the WGS84 geodetic system).

Species	Sample reference number	Locality	Date	Latitude, Longitude	Altitude m a.s.l.	Habitat	WD (m)	EC [µS/cm]	WT [°C]	pH	DO [mg/l]	alk [mmol/l]
**He**	NC-0919	Nam Co, STP	13.09.2019	30°47.40'N, 90°57.60'E	4728	Pond	0.2	ND	ND	ND	ND	ND
**He, Hi, Hs**	TIP11-84	Taro Co, STP	21.09.2011	31°11.11'N, 84°19.81'E	4577	Pond	ND	1342	10.1	9.2	3.5	10.7
**Hi**	HI-M19	San Nicolas Tetelco, Mexico City	24.03.2021	19°12.41'N, 98°58.24'W	2271	Flowerpot	0.1	ND	ND	ND	ND	ND
**Hi**	TIP08-4	Luo Ma town, STP	08.09.2008	31°16.49'N, 91°48.48'E	4587	Small lake	0.1	227	12.9	8.8	5.2	0.2
**Hi**	TIP11-99	Taro Co, STP	23.09.2011	31°19.42'N, 84°19.40'E	4578	Pond near inflow	0.2	236	16.6	9.4	2.9	1.4
**Hs**	HS-G19	Braunschweig, Germany	26.03.2021	52°16.23'N, 10°31.95'E	73	Flowerpot	0.2	ND	ND	ND	ND	ND
**Hs**	TIP11-86	Taro Co, STP	21.09.2011	31°11.21'N, 84°18.21'E	4588	Pond	0.1	1017	16.2	9.5	5.8	7.1
**Hs**	TIP12-H55	Pond near Xuru Co, STP	23.06.2012	30°10.17'N, 86°26.37'E	4728	Pond	0.1	1639	22.5	8.6	10.4	15.5

The Tibetan lakes are generally endorheic and strongly influenced by solar radiation, the Indian Summer Monsoon, and the Westerlies (Zhu et al. 2008). Winters are dry, and precipitation occurs mainly from May to September (Anslan et al. 2020). The local surrounding of the lake exhibits an average annual air temperature of 0 °C (Wang et al. 2020) and an estimated mean annual precipitation of 406 mm (Anslan et al. 2020). Furthermore, Gou et al. (2017) noted ice cover from early January through April in the lake’s vicinity and in Nam Co itself.

## ﻿Materials and methods

### ﻿Sampling and material handling

Ostracods were collected from surface sediments of a temporary pond near Nam Co, using a spatula and a hand net with a mesh size of 125 µm (Echeverría-Galindo et al. 2021; Vences et al. 2024). The material used to describe the new species corresponds to specimens previously referred to as Heterocypris aff. salina by Vences et al. (2024) and as Heterocypris cf. salina by Echeverría-Galindo et al. (2021). In both studies, these names were used provisionally due to the lack of a formal species description. In total, we examined material from eight sites (mainly in ponds), including samples from Nam Co collected in 2019, and more recent specimens from Mexico City (2021) and Braunschweig, Germany (2021) (see Table 1). Additionally, we included females of *H. exodonta* sp. nov., *H. incongruens*, and *H. salina* collected from other regions of the Southern Tibetan Plateau. These specimens originate from unpublished studies conducted in the vicinity of Luo Ma, Taro Co, and Xuru Co. Locations and environmental data of the samples are given in Table 1. To verify taxonomy, we decided to compare the new species with morphologically similar species previously recorded from the STP.

A total of ten adult females of each species were dissected using a stereo microscope (Leica MZ75); the right and left valves of each specimen were photographed to record their color. Female genital lobes were photographed with a Keyence^TM^ microscope (VHX-E100; objective ×100–×500). The valves were then placed onto micropaleontological slides, and subsequently photographed using scanning electron microscopy (SEM, ZEISS^TM^ EVO Ls 25) at the Institute for Chemical and Thermal Process Engineering, Faculty of Mechanical Engineering, TU Braunschweig. The material from the STP will be deposited at the Nanjing Institute of Geology and Palaeontology, Chinese Academy of Sciences, China. The material of *H. incongruens* from Mexico will be deposited at the National Collection of Crustaceans, Institute of Biology, National Autonomous University of Mexico. The material of *H. salina* collected in Braunschweig will be deposited at the Zoological Museum, Hamburg, Germany (**ZMH**).

Terminology and chaetotaxy of the limbs follow Broodbakker and Danielopol (1982), and Meisch (2000), except for the most posterior appendage (uropodal ramus) which follows Meisch (2007). Chaetotaxy of the antenna follows Horne (2005), and systematics refers to Meisch et al. (2024).

### ﻿Molecular analysis and phylogenetic methods

Genetic material extracted from the following four species was used: *Cyprinotus cassidula* Smith & Chang, 2020 (as a reference outgroup), *Heterocypris exodonta* sp. nov., *H. incongruens*, and *H. salina* (Table 2). Lysis buffer was prepared following established protocols for nematodes (Williams et al. 1992). All PCR reactions were conducted in a 25 μl volume, comprising 5 μl of DNA template, 2.5 μl of 10 × ExTaq Buffer, 0.25 μl of TaKaRa ExTaq (5 units/μl), 2 μl of dNTP Mixture (2.5 mM each), 1 μl of each primer, and 13.25 μl of distilled water. Partial sequences of mitochondrial COI were amplified using the following PCR protocol: initial denaturation at 94 °C for 5 min, followed by 35 cycles at 94 °C for 30 s, at 46 °C for 30 s, at 72 °C for 1 min, and a final extension at 72 °C for 10 min. This gene was amplified with universal Folmer primers (Folmer et al. 1994). The presence of DNA was confirmed using 1% agarose gel electrophoresis. After a treatment with LaboPass PCR Purification Kit (Cosmo Genetech), the PCR products were sequenced using an ABI automated capillary sequencer (Macrogen, Seoul, South Korea), employing the same sets of primers. Finch TV 1.4.0 (http://www.geospiza.com/Products/finchtv.shtml) was used to assess the quality of signal and to identify sites with potential low resolution, which were corrected by comparing forward and reverse strands. BLAST algorithm (Altschul et al. 1990) was used to check the identity of obtained sequences. All sequences were deposited in GenBank, and the accession numbers are listed as terminals on the phylogenetic trees.

**Table 2.** Sampling sites of *Heterocypris* and *Cyprinotus* species (He = *Heterocypris exodonta* Bonilla-Flores & Karanovic, sp. nov., Hi = *Heterocypris incongruens* (Ramdohr, 1808), Hs = *Heterocypris salina* (Brady, 1868), Cc = *Cyprinotus cassidula* (Smith & Chang, 2020) were used for molecular analysis. n: number of specimens, ND: no data.

Species	Sample reference number	Locality	Date	Latitude, Longitude	Altitude (m a.s.l.)	Habitat	Water dept (m)	n	Sex
***Cc***	CKF	Seonjudo park, South Korea	08.2022	37°54.00'N, 126°54.00'E	165	ND	ND	5	Female
***Cc***	CKF	Seonjudo park, South Korea	08.2022	37°54.00'N, 126°54.00'E	8	ND	ND	2	Male
***He***	NC-0919	Nam Co, STP	13.09.2019	30°47.40'N, 90°57.60'E	4728	Pond	0.2	10	Female
***Hi***	HI-M19	San Nicolas Tetelco, Mexico City	24.03.2021	19°12.41'N, 98°58.24'W	2271	Flowerpot	0.1	12	Female
***Hs***	HS-G19	Braunschweig, Germany	26.03.2021	52°16.23'N, 10°31.95'E	73	Flowerpot	0.2	12	Female

The obtained sequences, with those retrieved from GenBank, were aligned using MEGA11 (Tamura et al. 2021). The evolutionary model was tested using the IQ-TREE Web Server (Trifinopoulos et al. 2016), applying Akaike information criterion (Akaike 1974). Bayesian Inference, implemented in BEAST v. 2.5 (Bouckaert et al. 2014), was used to estimate phylogenetic relationships. The analysis ran for 10 million generations, sampling every 1000 generations. Tracer (Rambaut et al. 2018) was used to visualize the results of BEAST analyses, and FigTree v. 1.4.3 (available from http://github.com/rambaut/figtree) was used for tree visualization.

## ﻿Results

### ﻿Taxonomic account

**Class Ostracoda Latreille, 1802**

**Subclass Podocopa Sars, 1866**

**Order Podocopida Sars, 1866**

**Suborder Cypridocopina Baird, 1845**

**Superfamily Cypridoidea Baird, 1845**

**Family Cyprididae Baird, 1845**

**Subfamily Cyprinotinae Bronstein, 1947**

#### ﻿Genus Heterocypris

Taxon classificationAnimaliaOstracodaCyprididae

Claus, 1892

9ADCB2BA-C13F-532E-A18E-C75E451E039D

##### Brief diagnosis.

(after Meisch 2000 and Karanovic 2012): valves asymmetrical, the left valve (LV) longer and higher, overlapping the right valve (RV). In the dorsal area, LV lacks a hump, or gibbosity, and the RV has margins bearing denticles (Victor and Fernando 1980; Martens et al. 2019). Antenna with natatory setae extending beyond the tips of terminal claws, maxillular palp two-segmented, with the third endite bearing two-segmented and serrated teeth. Walking leg (T2) with a small seta d1; cleaning leg (T3) with a seta at the tip of the segment transformed into a pincer organ; uropodal ramus well developed.

#### ﻿ Heterocypris exodonta

Taxon classificationAnimaliaOstracodaCyprididae

Bonilla-Flores & Karanovic sp. nov.

34BDF600-157A-591B-A700-7A17F92C8AE0

https://zoobank.org/C2AC540D-7614-4CEE-A80E-F53CCA9794B6

Figs 2, 3, 4, 5

Heterocypris aff. salina : Vences et al. 2024: 1–9.
Heterocypris cf. salina : Echeverría-Galindo et al. 2021: 982–996.

##### Type locality.

Temporary pond near Nam Co, Southern Tibetan Plateau.

##### Material examined.

***Holotype***: China • 1 female, Nam Co, Southern Tibetan Plateau, appendages mounted on a glass slide, and the valves stored in a micropaleontological slide. Size RV: length = 1214 µm, height = 652 µm; LV: length = 1220 µm, height = 690 µm. Echeverría-Galindo et al. (2021) leg. ***Paratypes***: China • 7 females, in addition, eleven RV and nine LV separated; same location as holotype. Size RV: length = 994–1322 µm, height = 544–732 µm; LV: length = 1001–1337 µm, height = 555–734 µm, each specimen’s soft body mounted on a glass slide; valves stored in micropaleontological slides.

##### Additional material.

China • 1 female collected from Taro Co, STP, site TIP11-84 (Table 1). Size RV: length = 1113 µm, height = 610 µm; LV: length = 1135 µm, height = 624 µm; P. Frenzel leg.

##### Diagnosis.

**Figure 2.**
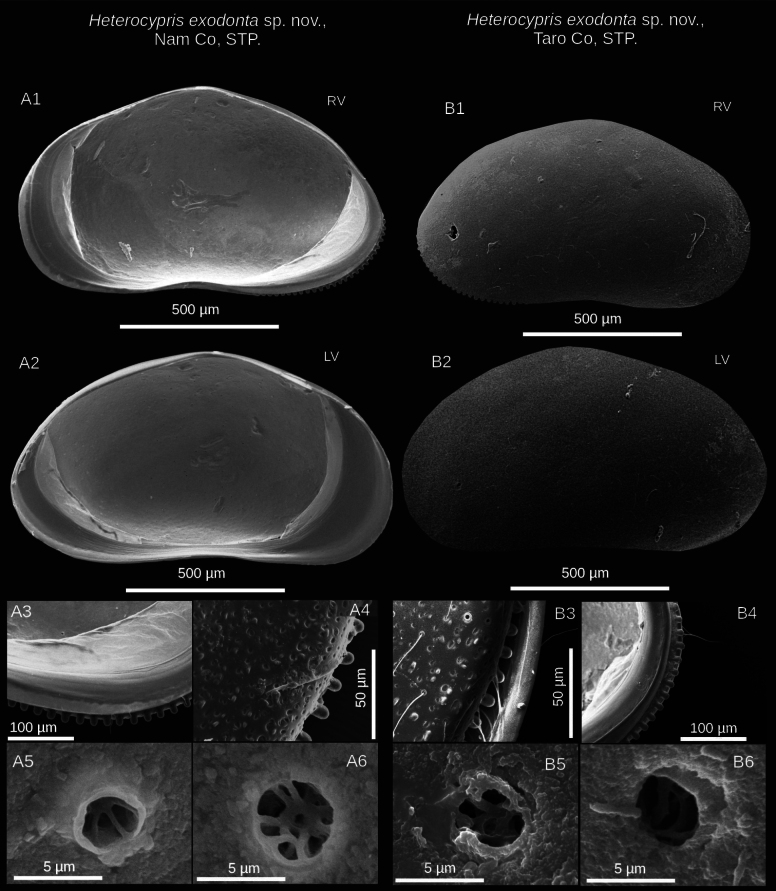
Valve details of *Heterocypris exodonta* Bonilla-Flores & Karanovic, sp. nov. from Nam Co (ID: NC-0919-E01) and Taro Co (ID: TIP11-84-E01), Southern Tibetan Plateau. Nam Co specimens: A1. RV and A2. LV lateral internal view of adult specimens. A3. Posterior internal view of RV; A4. Close-up of posterior external view of RV, with denticles in the margin. A5, A6. Close-ups of normal pores (turbine shape) without bristles and lip, in the central-internal region of RV. Taro Co specimens: B1. RV and B2. LV in lateral external view. B3. Anterior external view with denticles on RV; B4. Posterior internal view with denticles on RV. B5. Pore (turbine shape without bristle) in internal view, and B6. Simple pore in the central-internal region of RV.

**Figure 3.**
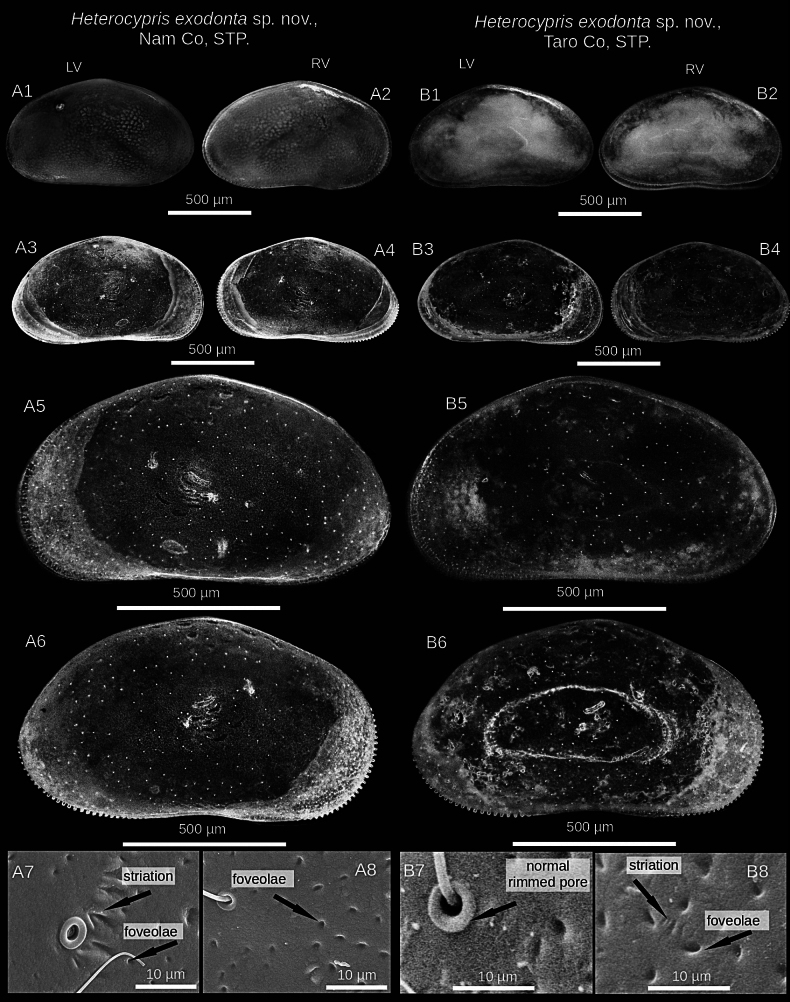
Microscopic and SEM images of the valves of *Heterocypris exodonta* Bonilla-Flores & Karanovic, sp. nov. from Nam Co (ID: NC-0919-E01) and Taro Co (ID: TIP11-84-E01), Southern Tibetan Plateau. Nam Co: A1. LV and A2. RV in lateral external view (whole carapace). A3. LV and A4. RV in lateral internal view. A5. LV and A6. RV in external view. A7, A8. Normal rimmed pores, striation, and foveolae in the external surface of RV. Taro Co: B1. LV and B2. RV lateral external view (whole carapace). B3. LV and B4. RV in lateral internal view. B5. LV and B6. RV in lateral external view. B7, B8. Normal rimmed pore, striations, and foveolae on the external surface of RV.

(adult females, Figs 2, 3) Valves elongated in lateral view. LV overlaps RV on all free margins. It is distinguished by a flattened dorsal margin and a pronounced row of blunt denticles on the anterior and posterior margins of the RV. The inner lamella is broad, and the inner list is well-developed, particularly in the LV. On the internal side, normal pores with a turbine shape but without a bristle in the center. The exopod seta in A2 is relatively shorter than the length of the second endopodal segment. Upper lip has a broad lip with a patch of pseudochaetae present laterally, immediately above the mouth opening. The second endopodal segment in T2 is proportionally short and the claw h2 is long. General shape of lobes is elongated, medium width; curvature of lobes is smooth, continuous; shape of apex is truncate with retrograde beak.

##### Dimensions.

Female, RV: length = 994–1322 µm, height = 544–732 µm; LV: length = 1001–1337 µm, height = 555–734 µm.

##### Description of holotype.

**Valves**: Surface of both valves with numerous normal rimmed (type A2; Danielopol et al. 2018) pores, each carrying one seta, as well as striations, and foveolae (Fig. 3A7–A8, B7–B8). In lateral view, lacks a hump on the dorsal side. RV with broad inner lamella and anterior and posterior margins with a well-differentiated row of denticles blunt, with inner list along anterior margin. LV with broad inner lamella in internal view (Fig. 2A2, B2). Normal pores on inner side with turbine-shaped opening and without a bristle in the center (Fig. 2A5, A6, B5, B6). Color yellowish (Fig. 3).

**Figure 4.**
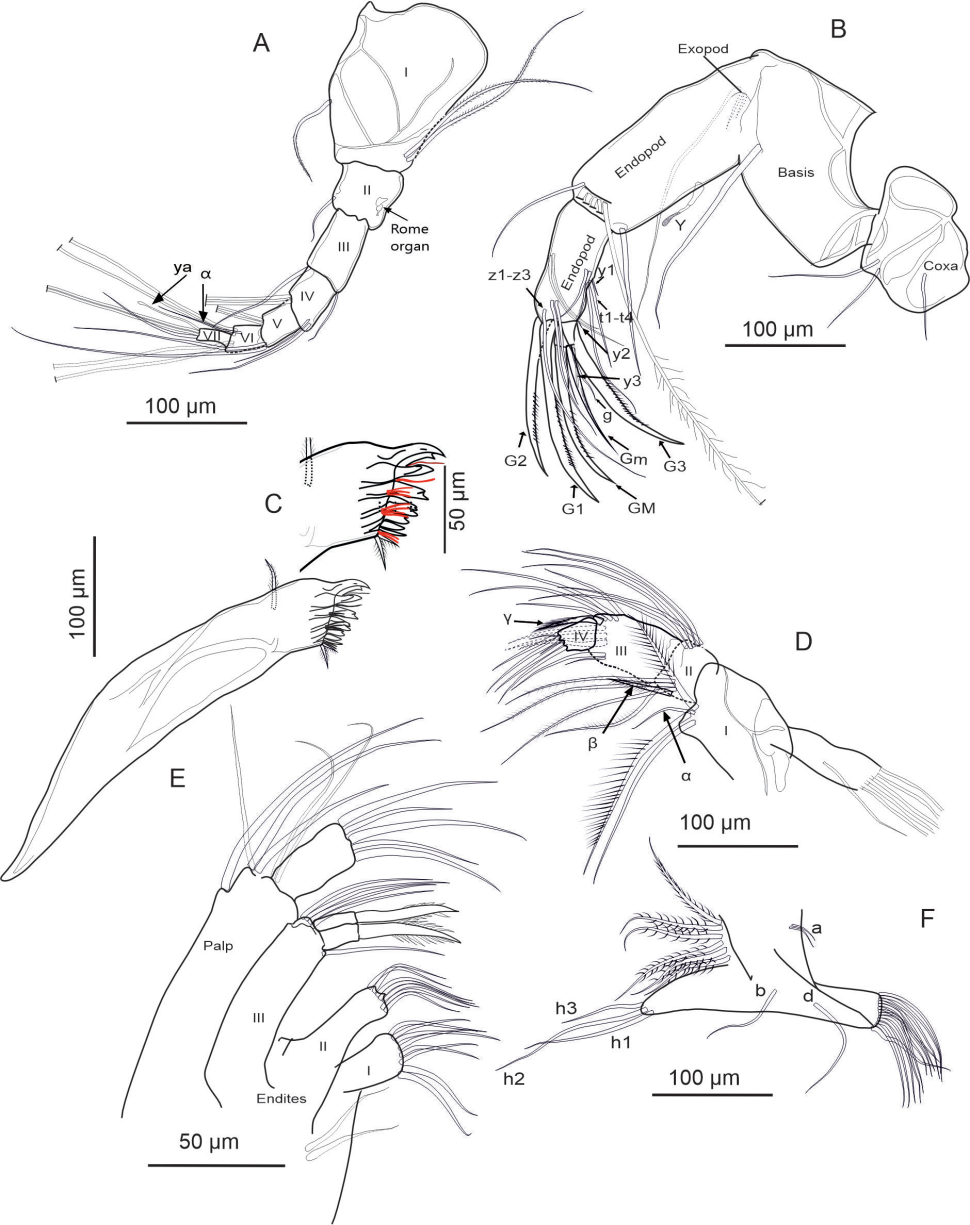
Holotype *Heterocypris exodonta* Bonilla-Flores & Karanovic, sp. nov. from Nam Co, Southern Tibetan Plateau (ID: NC-0919-E01), female. A. Antennule; B. Antenna; C. Mandibular coxa, showing a detail of the distal part; setae recognized between the teeth are highlighted in red. D. Mandibular palp; E. Maxillula; F. T1.

**Description of soft parts: *Antennule*** (Fig. 4A). Seven-segmented: segment I with three long posterodistal setose setae, and one short, smooth seta anteromedially. Segment II with one smooth distal seta anteriorly, and a Rome organ posteriorly. Segment III with one short, smooth distal seta on each side. Segment IV with four distal setae: two long setae located anteriorly, and two shorter, smooth setae spanning to segment VII. Segments V and VI with same chaetotaxy as segment IV, however, the ventral setae are longer. Additionally, segment VI bears a short alpha seta not present in the preceding segments. Segment VII with three setae distally (two long, one short; long ones setose, short one smooth), plus aesthetasc ya, which is 67 µm long, 2× longer than the terminal segment with 30 µm.

***Antenna*** (Fig. 4B). Five-segmented: coxa with three short setae; protopod or basis robust, with a smooth ventral distal seta. First endopodal segment with aesthetasc Y with distal dots; with five long swimming setae, and one small additional seta at the anterodistal side. Additionally, this segment includes one long setose seta posterodistally. Second endopodal segment with four t-setae (t1–t4), and aesthetascs y1, and y2, all situated posteromedially to posterodistally. Distally, this segment bears three long, thin z-setae and claws G1, G2, and G3. Claws G1 and G3 are approximately equal in length, while G2 is slightly shorter. Terminal segment with an aesthetasc y3 and claws GM and Gm; the former being more robust and slightly longer than the latter. Exopodal seta measures 205 µm in length, longer than the first (162 µm) and second (98 µm) endopodal segments. The second endopodal segment is 0.48× the length of the exopodal seta.

**Figure 5.**
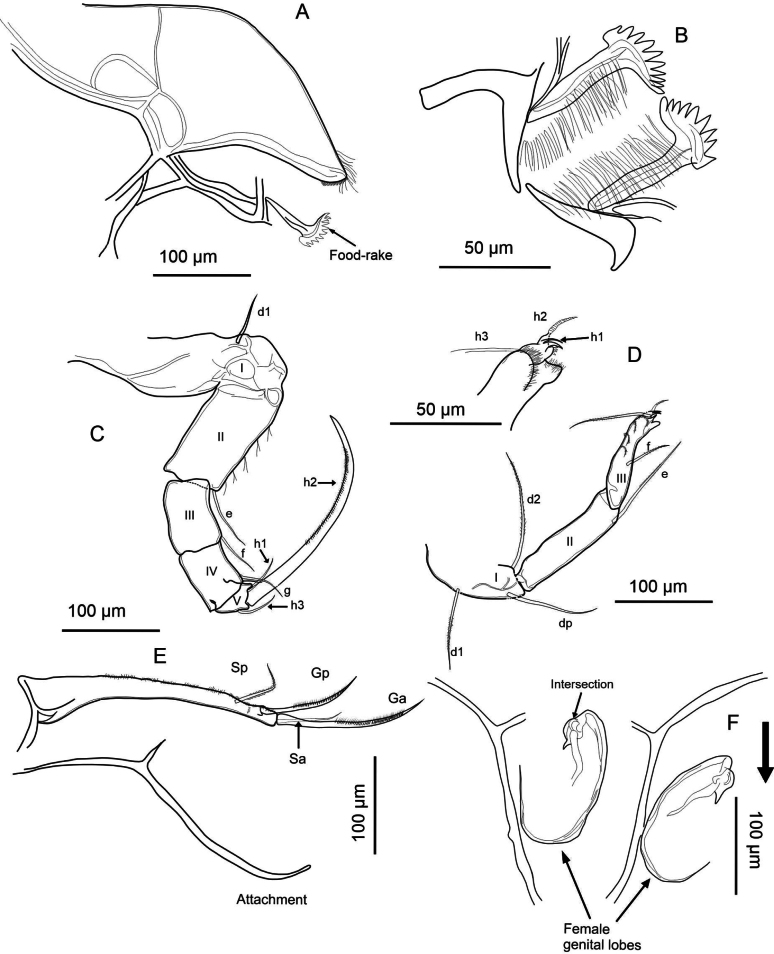
Holotype *Heterocypris exodonta* Bonilla-Flores & Karanovic, sp. nov. from Nam Co, Southern Tibetan Plateau (ID: NC-0919-E01), female. A. Upper lip; B. Rake-like organ; C. T2; D. T3 with detail of the tip; E. Uropodal ramus with attachment; F. Female genital lobes.

***Upper lip and rake-like organ*** (Fig. 5A, B). Measurements on the upper lip were taken as follows: l—length (263 µm), h—height (61 µm), hp—position of the maximum height (186 µm), in accordance with Karan-Žnidaršič and Petrov (2014). A large patch of dense pseudochaetae was present laterally, immediately above the mouth opening. Rake-like organ with nine teeth on the right and ten on the left.

***Mandibular coxa*** (Fig. 4C). With seven teeth, two setose distal setae, and one plumose seta situated subdistally. In addition, three groups of thick, short setae situated between teeth number three and four, and five, and close to inner end. One-seta between first and second, and second and third largest teeth.

***Mandibular palp*** (Fig. 4D). Four-segmented: first segment with respiratory plate (exopod) with six rays similar in length. Ventrally on the same segment, there are four setae: two plumose, one smooth, and one short, thin α-seta. The second segment carries ventrally a group of four long setae and a β-seta that is short, relatively thin, and setose. Dorsally on the same segment there are five long, smooth setae. Penultimate segment with nine smooth setae, and short, thick, setose, γ-seta. Segment IV with six smooth setae distally, two of which are short and thin.

***Maxillula*** (Fig. 4E). Maxillular palp two-segmented: first segment with six long, smooth setae, while second segment cylindrical featuring five smooth setae. The third endite of the maxillula palp features two serrated Zahnborsten. We identified five setae on endite 3, seven on endite 2, and eight on endite 1.

***T1*** (Fig. 4F). Endopod with three smooth, unequal setae, situated apically. Exopod (branchial plate) with six hirsute rays; protopod with 13 smooth setae. Two setae a short, setae d and b are present.

***T2*** (Fig. 5C). Five-segmented: incompletely divided protopod and five-segmented endopod. Basal segment with seta d1. First endopodal segment with seta e, overpassing the following segment. Second segment with seta f and third with a small seta near seta g. Terminal segment with short h1 and h3 setae, and h2 transformed into finely serrated, distally curved claw. The length of the second endopodal segment measures 10 µm, representing 51% the length of the claw, which measures 203 µm.

***T3*** (Fig. 5D). Four-segmented: basal segment with d1, d2, and dp setae present. Second segment bears a long distal e seta, while the last segment has a slightly shorter f seta. In the distal region of the last segment (fused 4^th^ segment), with its setae transformed into a pincer-shaped organ (all setae short except h3).

***Uropodal ramus*** (Fig. 5E). Elongated with two distal claws, Ga (144 µm) exceeding Gp (98 µm) in length, and one shorter setose Sp (61 µm). Additionally, a seta Sa is present and two simple caudal ramus attachments are observed.

***Female genital lobe*** (Fig. 5F). Lobe inverted in Fig. 5F to better represent shape. Incomplete crescent shape, with a vaginal opening displaying a tubular intersection. General shape of lobes is elongated, medium width; curvature of lobes is smooth and continuous; shape of apex is truncate with a retrograde beak.

##### Etymology.

From the Greek *exo* (outside) and *donta* (tooth); in relation to the well-defined crenulated teeth (pustules) observed along the anterior and posterior margins of the right valve (Fig. 2A1, A3, A4, B1, B3, B4).

##### Reproduction.

Possibly asexual.

##### Distribution.

Nam Co and Taro Co, STP.

##### Habitat.

This species was collected from surface sediments in a temporary pond with aquatic vegetation. According to limnological data (Table 1), this species inhabits fresh, alkaline, and low oxygenated waters (3.5 mg/l).

#### ﻿ Heterocypris incongruens

Taxon classificationAnimaliaOstracodaCyprididae

(Ramdohr, 1808)

90F3767E-B81E-5FF4-95B3-A1EC6601795B

Figs 6, 7, 8, 9

Cypris incongruens Ramdohr, 1808: figs 1–12, 15, 16, 19, 20.
Cypris fusca Straus: Fischer 1851: 156–157, pl. VIII, figs 9–13, pl. IX, figs 1–6, pl. XI, figs 10–12.
Cypris incongruens Ramdohr: Brady 1868: 362–364, pl. XXIII, figs 16–22.
Cypris incongruens Ramdohr: Vávra 1891: 95–98, fig. 32.
Heterocypris incongruens (Ramdohr), comb. nov.: Claus 1892: 161.
Heterocypris incongruens (Ramdohr): Sars 1924: pt. II, p. 116–117, pl. IV, figs 1, 2.
Heterocypris incongruens (Ramdohr): Poulsen 1940: 54–56, fig. 22.
Heterocypris incongruens (Ramdohr): Hartmann 1964: 33–34.
Heterocypris incongruens (Ramdohr): Meisch 2000: 346–351, figs 145, 146A–E.

##### Material examined.

Mexico • 10 females (size RV length = 1301–1404 µm, height = 740–803 µm; LV length = 1318–1455 µm, height = 805–853 µm); San Nicolas Tetelco, Mexico City; M. Bonilla-Flores leg. China • 1 female from Luo Ma, STP (TIP08-4) (size RV length = 1629 µm, height = 917 µm; LV length = 1647 µm, height = 958 µm); • 1 female from Taro Co, STP (TIP11-84) (size RV length = 1231 µm, height = 685 µm; LV length = 1252 µm, height = 697 µm); • twelve females from Taro Co, STP (TIP11-99) (size RV length = 1319–1405 µm, height = 741–826 µm; LV length = 1313–1336 µm, height = 768–807 µm); P. Frenzel leg.

##### Diagnosis.

**Figure 6.**
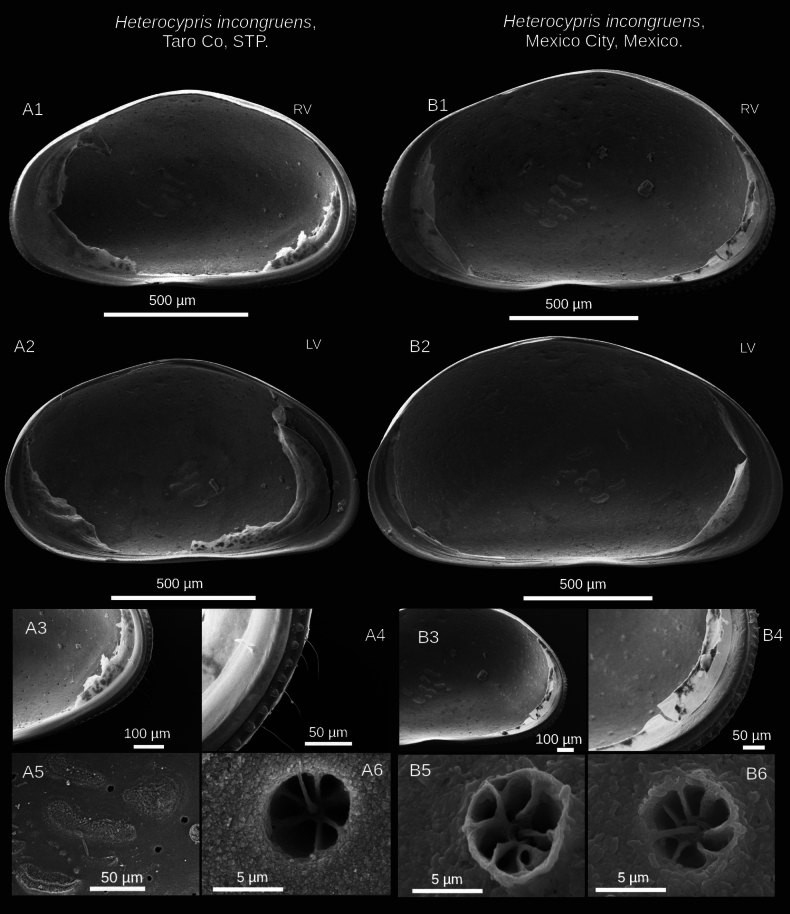
SEM images of valve details of *Heterocypris incongruens* (Ramdohr, 1808) from Taro Co, Southern Tibetan Plateau (ID: TIP11-84-I01), and Mexico City, Mexico (ID: HI-M19-I01). Taro Co: A1. RV and A2. LV of adult specimens, internal view. A3, A4. Denticles on the posterior region of the RV in internal view. A5. Adductor muscle scars and pores in internal view. A6. Close-up of a pore (turbine shape) with a bristle. Mexico City: B1. RV and B2. LV of adult specimens. B3, B4. Denticles in the posterior region of the RV in internal view. B5, B6. Pores (turbine shape) with a bristle, found in the central inner side of the RV.

**Figure 7.**
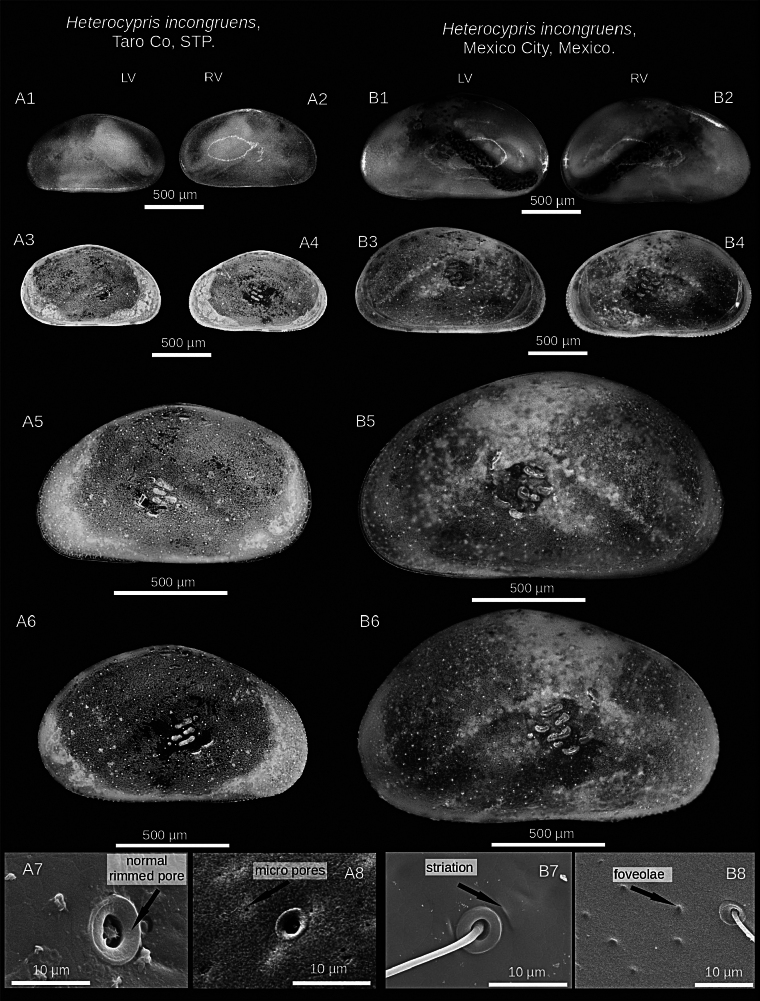
Microscopic image and SEM images of the valves of *Heterocypris incongruens* (Ramdohr, 1808) from Taro Co, Southern Tibetan Plateau (ID: TIP11-84-I01), and Mexico City, Mexico (ID: HI-M19-I01). Taro Co: A1. LV and A2. RV in external lateral view (whole carapace). A3. LV and A4. RV in internal lateral view. A5. LV and A6. RV in external lateral view. A7, A8. Normal rimmed pores on the external surface of RV. Mexico City: B1. LV and B2. RV in external view (whole carapace). B3. LV and B4. RV in internal view. B5. LV and B6. RV in external view. B7, B8. Surface shows striations, foveolae, and normal rimmed pores with a bristle on the external surface of RV.

(adult female, Figs 6, 7) (Meisch 2000): valves elongated in lateral view. RV is smaller than LV, the latter overlapping the right. The highest part is in the middle of the valves. RV with row of inconspicuous denticles in internal view on both margins, with a narrow inner lamella and inner list (Fig. 6A1, A3, A4, B1, B3, B4). LV without inner list (Fig. 6A2, B2). On the inner surface of the valve, there are normal, turbine-shaped pores with a bristle. The upper lip has a patch of medially positioned setae located in the middle region, approximately within the first third of the length from the mouth opening. Female genital lobes are subtriangular, the intersection displays a small beak-like projection, but the constriction is not pronounced. The lobes have a nearly straight distal margin.

##### Dimensions.

Adult females, RV ranges: length = 1136–1648 µm, height = 658–942 µm; LV length = 1134–1671 µm, height = 686–953 µm.

##### Valves.

External surface with numerous normal pores (Fig. 7A5, A6, B5, B6), striations, foveolae in antero-dorsal zone. In lateral view, dorsal side is arched. RV features a thin inner lamella, small crenulated teeth on anterior and posterior margins. LV with a thin inner lamella in internal view. Normal pores on inner surface have a turbine shape with a bristle (Fig. 6A6, B5, B6), but on external surface, pores present bristles and a rim (Fig. 7A7, A8, B7, B8). Color yellowish.

##### Description of soft parts.

**Figure 8.**
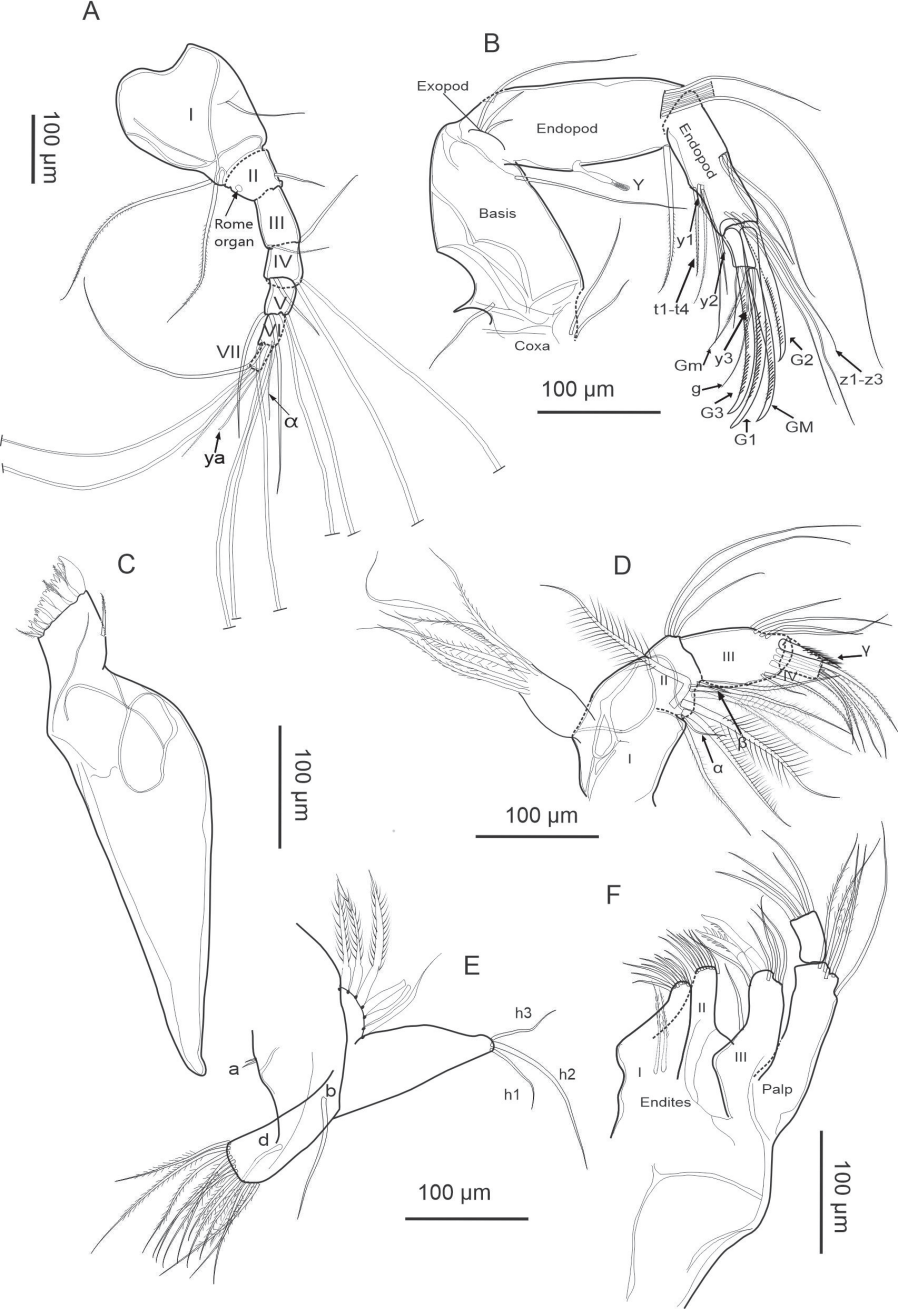
*Heterocypris incongruens* (Ramdohr, 1808), female, Mexico City (ID: HI-M19-I01). A. Antennule; B. Antenna; C. Mandibular coxa; D. Mandibular palp; E. T1; F. Maxillula.

**Figure 9.**
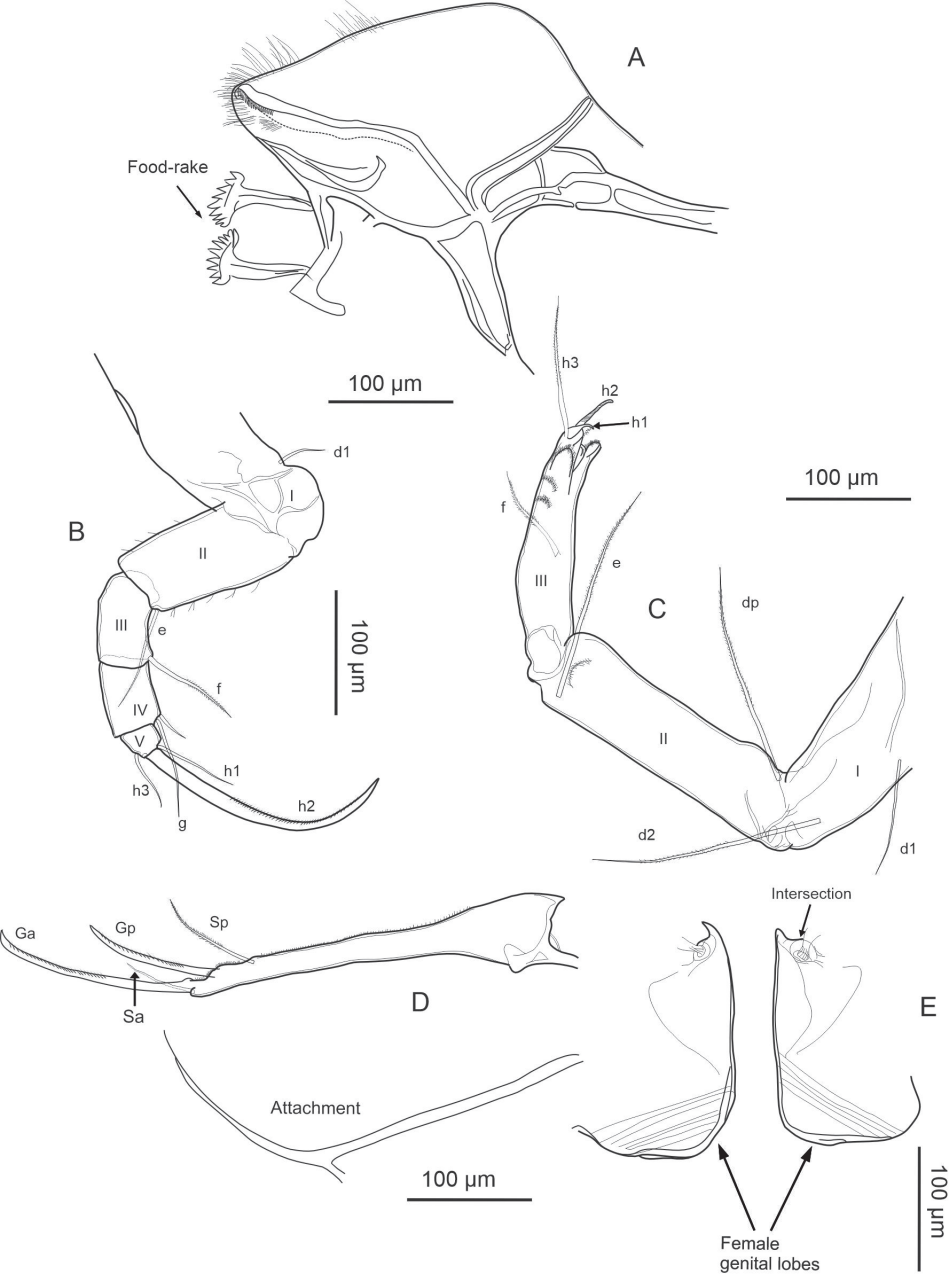
*Heterocypris incongruens* (Ramdohr, 1808), female from Mexico City, Mexico (ID: HI-M19-I01). A. Upper lip and rake-like organs; B. T2; C. T3; D. Uropodal ramus with attachment and E. Female genital lobes.

(Figs 8, 9) ***Antennule*** (Fig. 8A). Seven-segmented: segment I bears two long, setose setae on the posterodistal side and a short seta on the anterodorsal region. Segment II possesses a short seta on the anterior side. Segment III carries two short setae distally. Segment IV with three setae: two long ones on the anterior region and one short seta extending beyond segment V. Segment V features four setae, with those on the ventral side being longer. Segment VI bears a distal α-seta and four long setae. Segment VII with three distal setae, two long and one short. One aesthetasc ya, measuring 103 µm (twice the length of the terminal segment, 45 µm long).

***Antenna*** (Fig. 8B). Five-segmented: coxa with three short setae (illustrated incompletely); robust basis, with one long ventral seta. Exopod with three setae; two short ones and a long one. The first endopodal segment bears a two-segmented aesthetasc (Y) and five long swimming setae in the apical part, reaching to the tip of the claws, and one short seta. The second endopodal segment has four t-setae (t1–t4) and two aesthetascs (y1 in the posteromedial part and y2 in the posterodistal part). Distally, this segment bears three long, thin z-setae and three claws (G1, G2, and G3). Claws G1 and G3 are approximately equally long, while G2 is shorter. The terminal segment features an aesthetasc (y3), one g seta, and claws (GM and Gm). Exopod longest seta measures 147 µm in length, longer than the first (153 µm) and second (106 µm) endopodal segment; the length ratio of the second endopodal segment is 0.72× that of the exopod longest seta.

***Upper lip*** (Fig. 9A) has a distinctive internal reticulation. Measurements on the upper lip were taken as follows: length (290 µm), height (77 µm), position of the maximum height (208 µm), in accordance with Karan-Žnidaršič and Petrov (2014). A patch of medially positioned setae is in the middle region, approximately within the first third of the length from the mouth opening. Rake-like organ with nine teeth on the right and ten on the left.

***Mandibular coxa*** (Fig. 8C). With seven teeth and one plumose subdistal seta.

***Mandibular palp*** (Fig. 8D). Four-segmented: first segment bears a respiratory plate with six rays of similar length, plus three long distal setae, and an α seta. Second segment with a β seta, with four additional setae, including three long distal ones. Third segment features a γ seta on the apical part, and this same segment has nine setae in the distal region. Fourth segment features five setose setae, each measuring twice the length of the segment.

***Maxillula*** (Fig. 8E). Palp two-segmented: the first one bears a long, smooth seta with four setose setae, and the second segment is quadrangular in shape with five smooth long setae. The third endite holds two serrated two serrated Zahnborsten, plus five distal and a subdistal setae. Second endite with eight setae, and first endite with nine distal setae and two setae with rays in the basal region.

***T1*** (Fig. 8F). With two setae a, one b, and one d at the endopod. Exopod with six hirsute rays; protopod with 13 setose distal setae.

***T2*** (Fig. 9B). Five-segmented: the first one with a short seta d1. The second segment bears a long distal seta (e). The third segment has a setose distal seta (f). Segment four possesses a distal long seta (g) with a shorter seta. Segment five with short setae h1 and h3 and a claw-like seta h2 with spinules. The length of the second endopodal segments measures 108 µm, representing 59% of the length of the claw, which measures 183 µm.

***T3*** (Fig. 9C). Four-segmented: first segment bears a short, smooth d1-seta. In the distal region, there is a long setose seta d2, and a seta dp as long as the third segment. Second segment features a setose distal seta (e). Third segment displays a medial f-seta, a reduced h1 seta in the distal part (fused 4^th^ segment), a segmented h2 seta, and a long h3 setose seta.

***Uropodal ramus*** (Fig. 9D). Elongated structure with two distal claws—Ga (174 µm), exceeding Gp (122 µm) in length—and one shorter setose Sp (94 µm). A Sa seta is present, shorter than Sp, and two caudal ramus attachments are observed.

***Female genital lobes*** (Fig. 9E) have an intersection with a triangular shape. General shape of lobes is elongated and relatively broad; curvature of lobes is more open and straighter; shape of apex is pointed, without truncation.

##### Reproduction.

Commonly asexual populations (Meisch 2000), with records of males from a man-made pond, Kahramanmaraş, Turkey (Yavuzatmaca and Külköylüoğlu 2019), and several sexual records in Europe (Horne and Martens 1999)

##### Distribution.

Recorded from numerous places, it is cosmopolitan (Meisch 2000).

##### Habitat.

The species predominantly inhabits temporary ponds with vegetation cover (Meisch 2000; Mischke 2012). In accordance with our limnological data (Table 1), this species was found in freshwater to brackish, alkaline to oxygenated waters (average 3.8 mg/l). Ganning (1971) found this species up to a salinity of 16 psu at the Baltic Sea coast.

#### ﻿ Heterocypris salina

Taxon classificationAnimaliaOstracodaCyprididae

(Brady, 1868)

09BE9418-C98A-5639-AEC3-84CEEF724405

Figs 10, 11, 12, 13

Cypris salina Brady, 1868: 368–369, pl. 26, figs 8–13.
Cyprinotus fretensis Brady & Robertson, 1870: figs 48, 49 (Meisch and Broodbakker 1993).
Cyprinotus salina (Brady, 1868): Müller 1900: 76, pl. 16, figs 1, 2, 10, 12.
Heterocypris salina (Brady, 1868), nov. comb.: Klie 1932: 588.
Cyprinotus salinus (Brady, 1868): Wagner 1957: 30–31, pl. 9, figs 1–6.
Cyprinotus salinus (Brady, 1868): Jordan et al. 1962: 76, 77, pl. I, figs 6, 7; pl. V, figs 57–61; pl. VIII, figs 80, 82, 83.
Cyprinotus salinus (Brady, 1868): Devoto 1965: 332, fig. 26.
Cyprinotus salinus (Brady, 1868): Diebel and Pietrzeniuk 1975: 1213, pl. VII, figs 5, 6.
Cyprinotus salinus (Brady, 1868): Diebel and Pietrzeniuk 1978: 90, pl. 24, figs 5, 6.
Heterocypris salina salina (Brady, 1868): Freels 1980: 28, pl. 3, figs 1–6.
Heterocypris salina (Brady, 1868): Meisch 2000: 354–357, fig. 148.

##### Material examined.

Germany • 10 dissected females, (size RV length = 1012–1107 µm, height = 629–652 µm; LV length = 1098–1133 µm, height = 671–694 µm). Botanical Garden, TU Braunschweig; M. Bonilla-Flores leg. China • 2 females from Taro Co, STP (TIP11-84) (size RV length = 1068–1061 µm, height = 660–667 µm; LV length = 1079–1092 µm, height = 741–741 µm); • 2 females from Taro Co (TIP11-86) (RV length = 1011–1146 µm, height = 633–732 µm; LV length = 1024–1151 µm, height = 616–693 µm); • 1 female from Xuru Co, STP (TIP12-H55) (size RV length = 960 µm, height = 569 µm; LV length = 983 µm, height = 618 µm); P. Frenzel leg.

##### Diagnosis.

**Figure 10.**
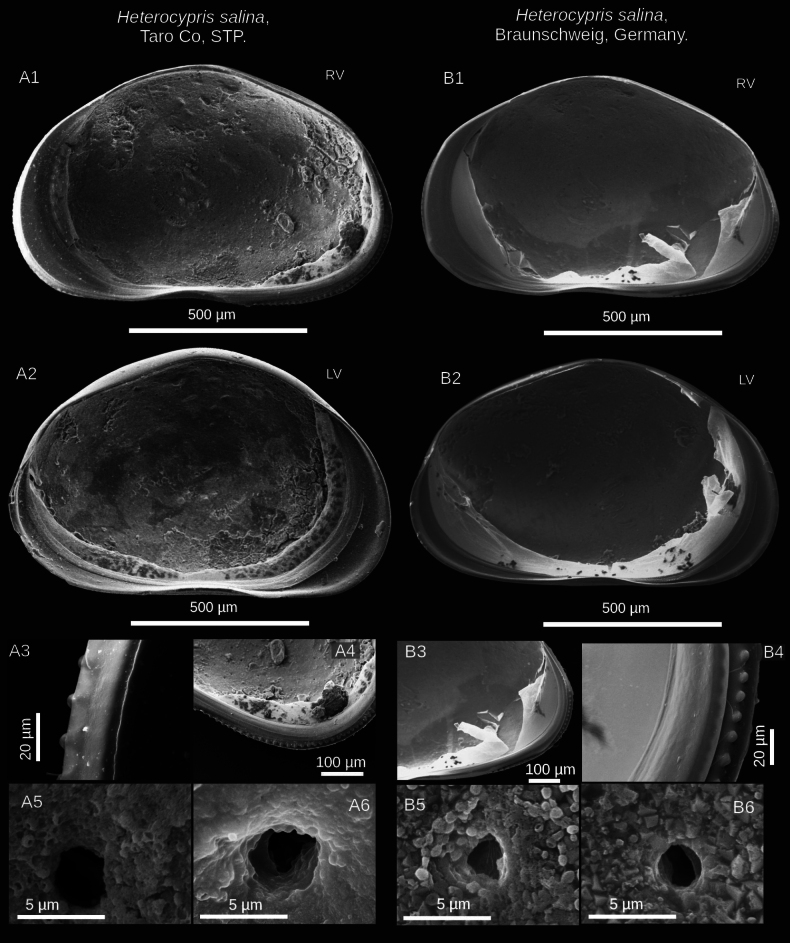
SEM images of valve details of *Heterocypris salina* (Brady, 1868) from Taro Co, Southern Tibetan Plateau (ID: TIP11-84-S01), and Braunschweig, Germany (ID: HS-G19-S01). Taro Co: A1. RV and A2. LV in internal view of adult specimens. A3. Denticles on the anterior margin, and A4. The posterior margin of the RV, internal view. A5, A6. Normal pores with simple openings located in the central inner region of the RV. Specimens from Braunschweig: B1. RV and B2. LV adult specimens, internal views. B3, B4. Denticles in the posterior region of the RV. B5, B6. Normal pores with simple opening located in the central inner area of the RV.

**Figure 11.**
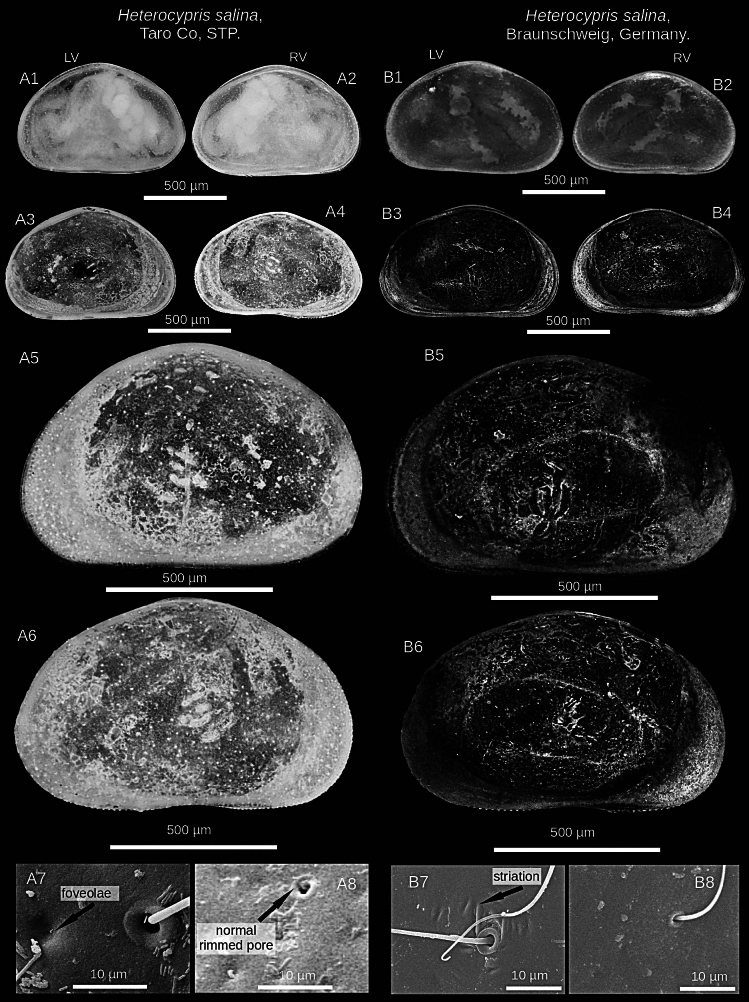
Microscopic image and SEM images of the valves of *Heterocypris salina* (Brady, 1868) from Taro Co, Southern Tibetan Plateau (ID: TIP11-84-S01), and Braunschweig, Germany (ID: HS-G19-S01). Taro Co: A1. LV and A2. RV, external view. LV (A3) and RV (A4), internal view. LV (A5) and RV (A6) in external view. A7, A8. Foveolae and normal rimmed pores with bristles on the external surface of RV. Specimens from Braunschweig: B1. LV and B2. RV, external view. B3. LV and B4. RV in internal view. B5. LV and B6. RV, external view. B7, B8. Show striations and normal rimmed pores with bristles on the external surface of RV.

(Adult female, Figs 10, 11) (adapted from Meisch 2000 and Kubanç et al. 2007): valves compressed and triangular in the dorsal region. RV smaller than LV, LV overlapping RV. RV has row denticles on anterior and posterior margins in external view, with broad inner lamella and inner list (Fig. 10A1, A3, A4, B1, B3, B4). LV with a broad inner lamella, anteriorly with inner list, in the posteromedial region with a slight fold (Fig. 10A2–B2). Both valves display a brown coloration pattern, characterized by a pair of lighter stripes (Fig. 11B1, B2). Normal pores on the internal valve surface with simple apertures. The upper lip presents dense pseudochaetae laterally, just above the mouth opening. Female genital lobes with an oval shape, with no discernible projection at the intersection, but the shape of the apex is truncated, and an interlacing projection at the intersection forms a ring.

##### Dimensions.

Adult females, RV ranges: length = 970–1194 µm, height = 577–755 µm; LV ranges: length = 989–1189 µm, height = 603–724 µm.

##### Valves.

Laterally, dorsal side arched. RV has a broad inner lamella with small denticles along the anterior and posterior margins. LV displays a broad inner lamella accompanied by an inner list. Normal pores on the internal valve surface with simple apertures (Fig. 10A5, A6, B5, B6). Similarly, multiple normal rimmed pores are present on the external valve surface, with a single bristle (Fig. 11A7, A8, B7, B8). Some striations were observed for organisms from Germany (Fig. 11B7).

##### Description of soft parts.

**Figure 12.**
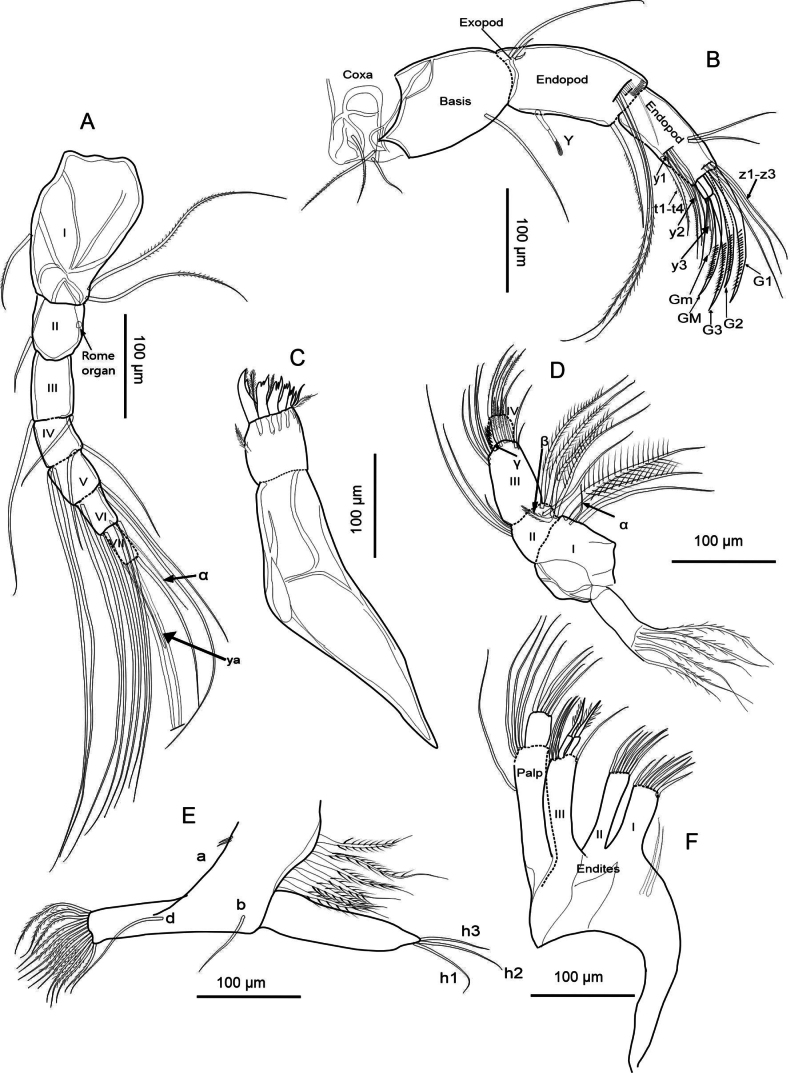
Female *Heterocypris salina* (Brady, 1868) from Braunschweig, Germany (ID: HS-G19-S01). A. Antennule; B. Antenna; C. Mandibular coxa; D. Mandibular palp; E. T1; F. Maxillula.

**Figure 13.**
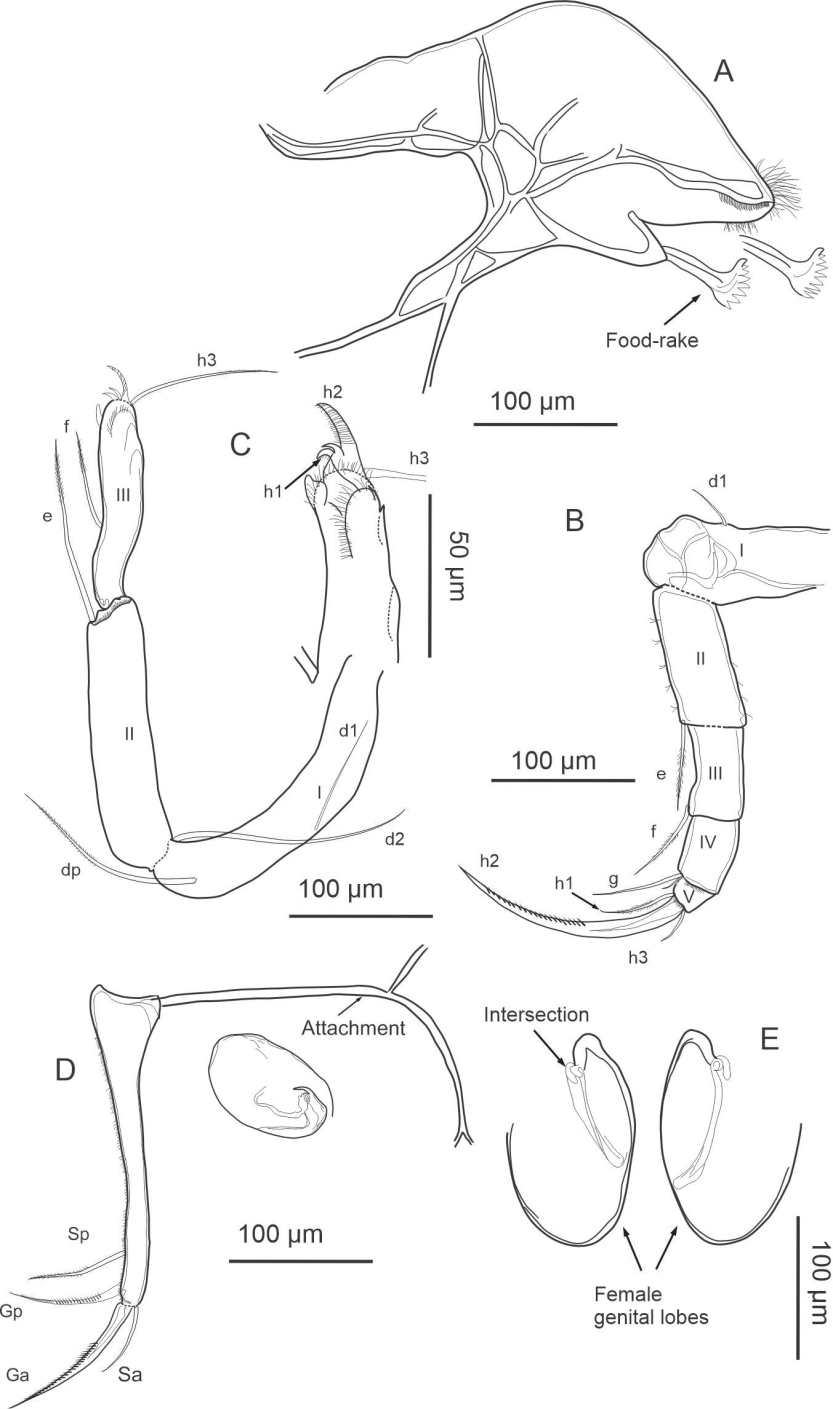
Female *Heterocypris salina* (Brady, 1868) from Braunschweig, Germany (ID: HS-G19-S01). A. Upper lip and rake-like organs; B. T2; C. T3 with detail of the tip; D. Caudal ramus with attachment, and E. Female genital lobes.

(Figs 12, 13): ***Antennule*** (Fig. 12A). Seven-segmented: segment I with two long setose setae on the posterodistal side and one short seta on the anterodorsal region. Segment II with a short seta on the anterior side. Segment III carries two distal setae, with the anterodorsal seta reaching segment VII. Segment IV bears two long anterodorsal setae and two short posterodistal setae. Segment V features four long setae in the distal region. Segment VI with an α-setae in the distal part, with four long setae. Segment VII presents three long setae distally and one aesthetasc ya with 65 µm length, roughly twice as long as the terminal segment (31 µm).

***Antenna*** (Fig. 12B). Five-segmented: coxa has two short hairy setae and one smooth short seta. Basis with a ventrodistal long seta. Exopod long seta as long as the first endopodal segment, which bears a segmented aesthetasc Y, a stout ventroapically seta, one short and five long natatory setae in the apical dorsal part. The second endopodal segment has four t-setae and two aesthetascs (y1 in the posteromedial part and y2 in the posterodistal part). This segment also bears two medial setae, and distally three long, slender setae (z) and three claws (G1, G2, and G3). Claws G1 and G3 are approximately equally long, while G2 is shorter. The terminal segment presents two claws (GM and Gm), and the aesthetasc y3. Exopod long seta (140 µm length) longer than the first (119 µm) and second (96 µm) endopodal segment, respectively; the exopod long seta is approximately 1.46× the length of the second endopodal segment.

***Upper lip*** (Fig. 13A). Measurements on the upper lip were taken as follows: l—length (245 µm), h—height (64 µm), hp—position of the maximum height (168 µm), in accordance with Karan-Žnidaršič and Petrov (2014). Dense pseudochaetae were present laterally, just above the mouth opening. Rake-like organs are formed by eight teeth on the right, and nine on the left.

***Mandibular coxa*** (Fig. 12C) with seven teeth, the largest one with small setae on its ventral part. Teeth three, four, and seven lack setae between them.

***Mandibular palp*** (Fig. 12D). Four-segmented: the first segment bears two setose setae and one smooth seta, and an additional α-seta. The second segment has four setose setae, one long smooth seta, and one short setose β-seta on the inner part, and three smooth setae on the outer part. The third segment features eleven setae; four long smooth setae, with one thick γ-seta. In the most distal region, there are three short, setose setae and three smooth setae. The last segment has three setose setae in the distal region and one smooth seta.

***Maxillula*** (Fig. 12E). Maxillular palp two-segmented, the first one bears a long, smooth seta with four long and smooth distal setae, the second segment of the palp is subrectangular with four smooth setae. First endite with eight setae in distal part, and two setae in the basal region. Seven setae in second endite. The third endite has two serrated Zahnborsten, plus five smooth setae.

***T1*** (Fig. 12F). With two setae a, one b, and one d. Exopod with six hirsute rays, protopod with 13 setose distal setae. Endopod with three distal setae, h1 and h3 longer than h2.

***T2*** (Fig. 13B). Five-segmented: segment I with a short seta d1. Second segment bears a long anterodistal e-seta. Third segment with a distal setose f-seta. Segment four with a distally located g-seta with a shorter seta. Fifth segment with three h-setae; the claw-shaped seta h2 with dentition. The length of the second endopodal segment measures 95 µm, representing 61% of the length of the claw which measures 156 µm.

***T3*** (Fig. 13C). Four-segmented: first segment bears a short, smooth seta d1. In the distal region, with a long d2-seta, and a setose dp-seta. Second segment with a setose distal e-seta. Third segment in the distal part (fused 4^th^ segment) has a small h1-seta, seta h2 is striated, and seta h3 long.

***Uropodal ramus*** (Fig. 13D) with an elongated shape and two distal claws – Ga (137 µm), exceeding Gp (97 µm) in length – plus one shorter setose Sp (80 µm). Additionally, with a smooth Sa seta, shorter than Sp, and two caudal ramus attachments.

***Female genital lobes*** (Fig. 13E). Oval and elongated shape, curvature of lobes; shape of apex is truncated, without a beak, and an interlacing projection at the intersection forms a ring.

##### Reproduction.

Only asexual populations were found in Germany and STP. Males were recorded from Crete, Greece (Petkowski et al. 2000).

##### Distribution.

Widespread distribution (Meisch 2000).

##### Habitat.

*Heterocypris salina* is an eurytopic species, tolerant to high conductivity, typical of shallow and temporary ponds (Meisch 2000). Furthermore, according to our limnological data (Table 1) and previous publications, the species is found in fresh, brackish, and saline waters (Ganning 1971), and alkaline and oxygenated waters (average 6.5 mg/l). Upper salinity limits are given as 8.6‰ (Vesper 1975) and even up to 20‰ (Griffiths and Holmes 2000). It is also tolerant to organic pollution (Mezquita et al. 1999).

### ﻿Molecular analysis

**Figure 14.**
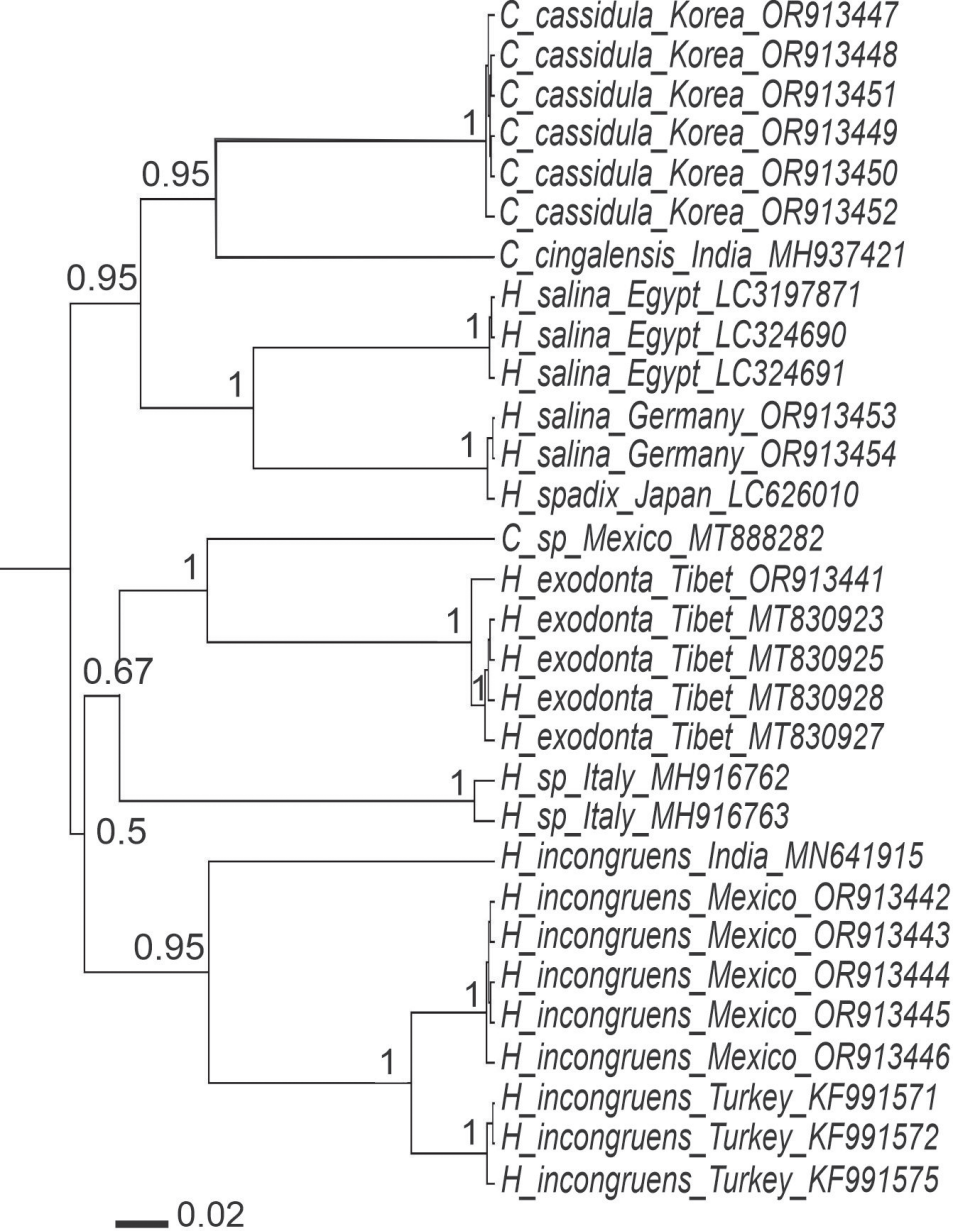
Bayesian Inference (BI) phylogenetic tree based on mitochondrial cytochrome c oxidase subunit I (COI) sequences of *Heterocypris exodonta* Bonilla-Flores & Karanovic, sp. nov., *Heterocypris incongruens* (Ramdohr, 1808), *Heterocypris salina* (Brady, 1868), *Heterocypris* sp. from Italy and *Cyprinotus* sp. from Mexico, *Heterocypris spadix* Munakata, Tanaka & Kakui, 2021, *Cyprinotus cassidula* Smith & Chang, 2020, and *Cyprinotus cingalensis* Brady, 1886. Node values represent posterior probabilities from BI analysis. Terminal labels indicate species, country of origin, and GenBank accession numbers. Sequences of *Heterocypris exodonta* sp. nov. with numbers MT830923, MT830925, MT830927, and MT830928 were registered as Heterocypris cf. salina in Echeverría-Galindo et al. (2021) and Vences et al. (2024). The scale bar represents 0.02 substitutions per site.

The COI alignment included 30 sequences, with 14 newly obtained (Code *OR91*) and the remainder downloaded from GenBank (Fig. 14). The alignment spanned 715 base pairs, but sequence lengths varied from 317 to 683 base pairs. Within group p-distances (Table 3) ranged from 1% (in *Heterocypris exodonta* sp. nov. and *Heterocypris* sp.) to 9% (in *H. salina* from Egypt and Germany). The largest between-group distances (Table 3) were observed between *H. exodonta* sp. nov. and *Cyprinotus cingalensis* Brady (1889), equaling 25%, and the smallest were between *H. salina* and *H. spadix* Munakata, Tanaka & Kakui, 2021, equaling 10%.

**Table 3.** Pairwise genetic distances (p-distances) based on cytochrome c oxidase subunit I (COI) sequences among species of *Heterocypris* Claus, 1892 and *Cyprinotus* Brady, 1886, calculated using 1000 bootstrap replications. Values represent average distances between groups; diagonal entries (in bold or along the row) indicate within-group variation. Sequences of *Heterocypris exodonta* Bonilla-Flores & Karanovic, sp. nov. are compared with closely related species and outgroups.

Species	*H.* sp.	*H. incongruens*	*H. salina*	*H. spadix*	*C.* sp.	*C. cingalensis*	*C. cassidula*
*H. exodonta* sp. nov.	0.01						
*H.* sp.	0.23	0.01					
*H. incongruens*	0.22	0.19	0.06				
*H. salina*	0.24	0.20	0.20	0.09			
*H. spadix*	0.24	0.21	0.20	0.10/			
*C.* sp.	0.23	0.20	0.20	0.21	0.22/		
*C. cingalensis*	0.25	0.21	0.21	0.19	0.21	0.20/	
*C. cassidula*	0.24	0.21	0.21	0.19	0.21	0.23	0

The JC69 (Jukes and Cantor 1969) model was selected as the most appropriate model for DNA evolution. In the resulting phylogenetic tree (Fig. 14), *H. exodonta* sp. nov. clustered with H. cf. salina sequences from the STP (Echeverría-Galindo et al. 2021; Vences et al. 2024), as they derive from the same material. However, to avoid further confusion, the name was changed to *H. exodonta* sp. nov. The tree suggests a sister relationship between the new species and *Cyprinotus* sp. from Mexico (GenBank number MT888282), but the p-distance between these two species is more than 20%, and there is the possibility that the Mexican species was misidentified and, thus, does not belong to *Cyprinotus* Brady, 1886.

Furthermore, the sister relationship between the new species *Heterocypris exodonta* sp. nov., *H.* sp. from Italy, as well as *H. incongruens* from India, Mexico, and Turkey received a low posterior probability (0.67 and 0.5). Although a monophyly of all *H. incongruens* specimens was strongly supported with a high posterior probability (0.95), p-distances between the Indian sequences and those from Mexico and Turkey were 17% and 18%, respectively. The p-distances between sequences from Mexico and Turkey were only 6%. The phylogenetic tree also suggests a sister relationship between *H. salina*, *H. spadix* Munakata, Tanaka & Kakui, 2021, and two *Cyprinotus* species (*C. cassidula* Smith & Chang, 2020, *C. cingalensis* Brady, 1886), suggesting a broader morphological range of the genus *Heterocypris*. The p-distance between *H. salina* from Germany and Egypt was also high (16%).

## ﻿Discussion

In the genus *Heterocypris*, the examination of appendages and valve characteristics constitutes the primary approach for distinguishing and comparing morphologically similar species (Meisch 2000). However, in species such as *H. incongruens* and *H. salina*, various synonymies arose due to their considerable phenotypic plasticity, wide distribution, cryptic speciation, and the absence of defining features enabling their clear differentiation (Martens et al. 2002; Kilikowska et al. 2024), which are discussed below.

### ﻿Morphological comparison between *Heterocypris exodonta* sp. nov., *Heterocypris incongruens* (Ramdohr, 1808), and *Heterocypris salina* (Brady, 1868)

**Figure 15.**
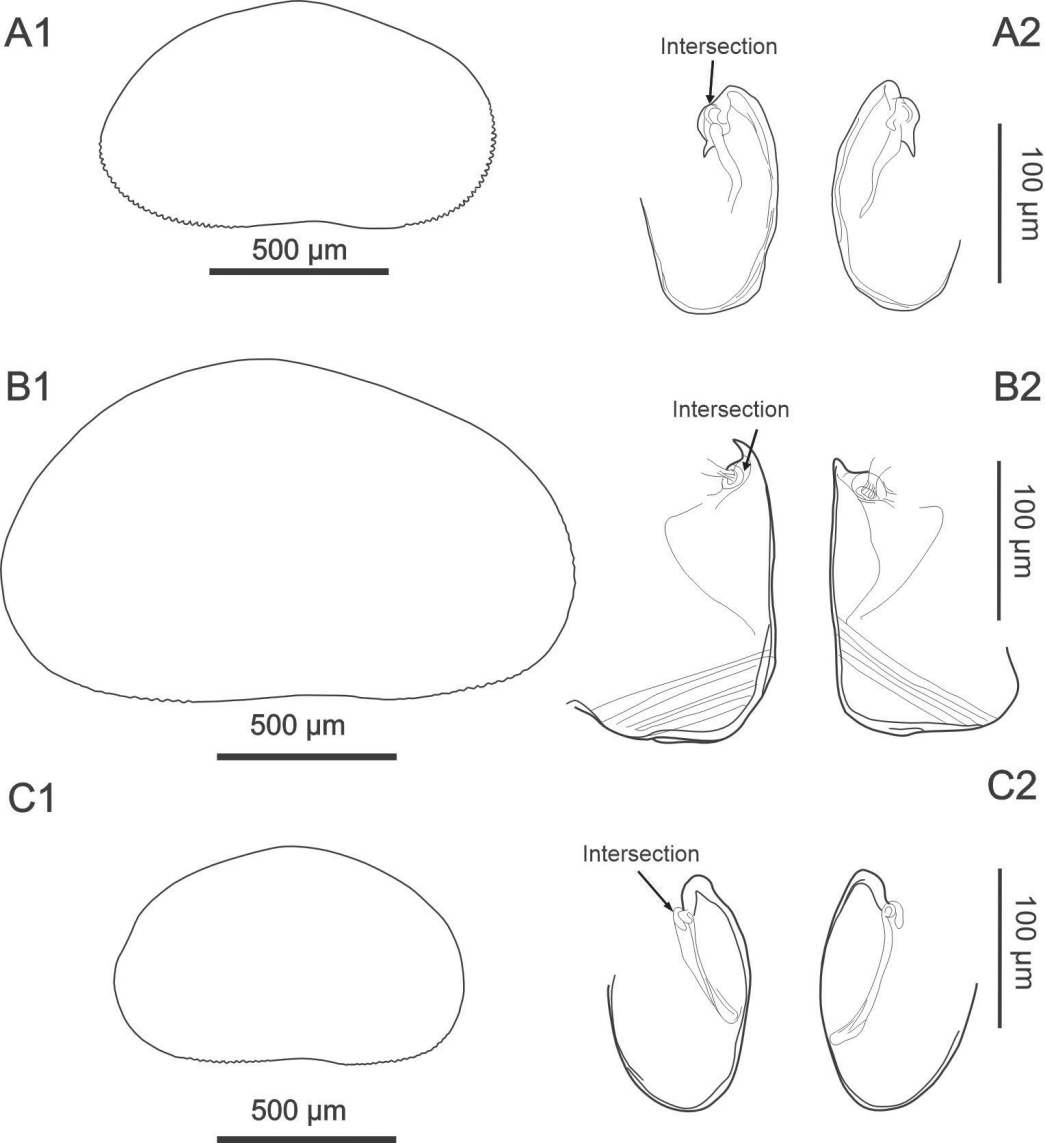
Comparison of the outlines of the right valves and female genital lobes in *Heterocypris exodonta* Bonilla-Flores & Karanovic, sp. nov. (A1, A2), *Heterocypris incongruens* (Ramdohr, 1808) (B1, B2), and *Heterocypris salina* (Brady, 1868) (C1, C2). In *Heterocypris exodonta* sp. nov., the dentition along the valve margins is more pronounced than in *Heterocypris incongruens* and *Heterocypris salina* (Brady, 1868).

The main differences between *H. exodonta* sp. nov., *H. incongruens*, and *H. salina* are the shape of the valves, dentation on the RV margin, and the shape of the female genital lobes (Fig. 15). The three species examined also differ in valve size, with *H. incongruens* being the largest and *H. salina* the smallest (Table 4). According to Meisch (2000), the average body length of female *H. salina* ranges from 800 to 1300 µm (commonly 900–1200 µm), while in *H. incongruens*, female lengths range from 1200 to 1900 µm (typically 1400–1600 µm), and males from 1200 to 1300 µm. Our specimens of *H. salina* fall within the lower range of these values, with average valve lengths of 1039 µm (RV) and 1045 µm (LV). Interestingly, Petkowski et al. (2000) reported larger *H. salina* females in Bosnia, reaching up to 1270 µm. In contrast, *H. exodonta* sp. nov. females show intermediate valve lengths (RV = 1130 µm; LV = 1143 µm), which exceed those recorded for *H. salina* from Germany (see Table 4), further supporting their distinctiveness. Additionally, our measurements of *H. incongruens* from Mexico (RV = 1349 µm; LV = 1360 µm) align with previously reported sizes for this species, confirming its relatively larger body size. Although there is some overlap in valve length among the three species, the observed differences, particularly when combined with other morphological traits and molecular data, underscore the diagnostic value of morphometric characters such as valve size. These findings highlight the importance of integrative approaches in ostracod taxonomy, where morphometric measurements contribute significantly to species delimitation.

**Table 4.** Average length and height, including standard deviation, of right (RV) and left (LV) adult valves of *Heterocypris exodonta* Bonilla-Flores & Karanovic, sp. nov. from Nam Co, STP, *Heterocypris incongruens* (Ramdohr, 1808) from Mexico City, Mexico, and *H. salina* (Brady, 1868) from Braunschweig, Germany.

Species	n	Length RV (µm)	Height RV (µm)	n	Length LV (µm)	Height LV (µm)
*H. exodonta*	20	1130 ± 105	628 ± 55	18	1143 ± 112	636 ± 57
*H. incongruens*	52	1349 ± 140	790 ± 75	57	1360 ± 146	788 ± 83
*H. salina*	110	1039 ± 64	622 ± 44	119	1045 ± 55	638 ± 37

Moreover, in *Heterocypris exodonta* sp. nov., the denticles are more prominent than in *H. incongruens* and *H. salina*. The dorsal margin of the RV in *H. exodonta* sp. nov. is elongated without a dorsal hump, while the dorsal margin of *H. salina* is strongly arched. The color of *H. exodonta* sp. nov. is yellowish and uniform for both valves, while the surface of both valves in *H. salina* is covered with fine setae and displays a brown color pattern, with two lighter stripes (Fig. 11B1, B2). In contrast, the valves of *H. incongruens* are uniformly yellowish in color (Table 5), as previously reported by Meisch (2000).

**Table 5.** Comparative morphological characteristics among *Heterocypris exodonta* Bonilla-Flores & Karanovic, sp. nov., *Heterocypris incongruens* (Ramdohr, 1808), and *Heterocypris salina* (Brady, 1868).

Species	Average size (µm) of RV	Shape of RV	Coloration in living individuals	Shape of female genital lobes	Conspicuous or inconspicuous margin crenulation of RV	Shape of the internal normal pores of the valves
*H. exodonta*	1130	Elongated	Yellowish	Incomplete crescent shape, intersection with apex truncated, bearing a retroflexed projection.	Conspicuous	Turbine without a bristle.
*H. incongruens*	1349	Elongated	Yellowish	Triangular shape, apex pointed.	Inconspicuous	Turbine with a bristle.
*H. salina*	1039	Strongly arched	Brown color pattern, with two lighter perpendicular lines	Oval shape, apex truncated, without a distinct projection, but at the intersection, forming a ring-like feature.	Inconspicuous	Simple opening

The morphology and relative lengths of the caudal ramus setae Sp and Sa differ among *Heterocypris exodonta* sp. nov., *H. incongruens*, and *H. salina*, and also in comparison with other cypridid species. In *H. incongruens*, the Sp seta is noticeably longer than the Sa seta, whereas in *H. exodonta* sp. nov. and *H. salina*, both setae are of similar length. This pattern contrasts with *H. bosniaca* (Petkowski et al. 2000; Aguilar-Alberola and Mesquita-Joanes 2013), in which the Sa seta is of comparable length to that of *H. salina* and *H. exodonta* sp. nov. Similarly, in *Heterocypris sarakhamensis* (Savatenalinton, 2020) and *Cyprinotus dubrea* (Martens, Yavuzatmaca & Higuti, 2019), the Sp and Sa setae are of approximately equal length, resembling the condition in *H. exodonta* sp. nov. and *H. salina*, and differing from *H. incongruens*, where the Sp seta is relatively longer.

A similar trend of interspecific variation is observed in the length of the d1 seta on the second thoracopod (T2). Among the three focal species, the d1 seta is longest in *H. exodonta* sp. nov. (53 µm), shorter in *H. incongruens* (38 µm), and shortest in *H. salina* (35 µm). In comparison, *H. bosniaca* exhibits a considerably shorter d1 seta (19 µm) (Petkowski et al. 2000; Aguilar-Alberola and Mesquita-Joanes 2013), while *H. sarakhamensis* and *C. dubrea* show intermediate lengths of 41 µm and 31 µm, respectively (Martens et al. 2019; Savatenalinton 2020). These comparative differences in seta morphology provide useful taxonomic characters for species-level identification within the *Heterocypris* genus and related cypridids.

The specific morphological traits of the genital lobes, such as shape, curvature, and apex configuration, provide essential diagnostic characters for species identification. In *H. exodonta* sp. nov., *H. incongruens*, and *H. salina*, differences in lobe elongation, width, curvature, and apex structure allow clear separation of otherwise similar taxa (Fig. 15). These features contribute to more accurate taxonomic keys and the study of intraspecific variability. Kong et al. (2014) used soft part morphology, including female genital lobes, to distinguish *Chrissia dongqianhuensis* Kong, Karanovic & Yu, 2014 from *Stenocypris major* (Baird, 1859), illustrating their diagnostic value. However, additional comparative studies across other genera are needed to verify the consistent usefulness of female genital lobes for species-level differentiation and phylogenetic relationships.

### ﻿Ultrastructure of pores

There are two main pore types in ostracods. (1) Normal pores consist of a simple hole, sometimes with a rim or lip, and (2) sieve pores, characterized by a sieve-like cover over the hole, mainly found on the surface of the valves (Karanovic et al. 2017; Danielopol et al. 2018; Smith 2023). Despite their importance, only few taxonomic studies have thoroughly described the shape and type of these structures (Meisch and Wouters 2004; Lord et al. 2020). Moreover, while variations in the shape of the normal pore openings on the inner side of the valves have been observed, there is little published data on this topic, especially for species within the family Cyprididae (Meisch and Wouters 2004).

For both *Heterocypris exodonta* sp. nov. and *H. incongruens*, we report for the first time normal pores displaying turbine-shaped openings on the inside surface of the valves (Figs 2A5, A6, B5, B6, 6A5, B5, B6). Conversely, for *H. salina*, only simple pores were observed, with round openings in internal view (Figs 10A5, A6, B5, B6). The specific function of these structures remains unknown, although some hypotheses suggest a possible relationship with osmoregulation, oxygenation, or excretion (Lord et al. 2020). It was noted that pore types and their ornamentation are influenced by environmental factors, particularly salinity (Hou and Zhao 1988). Additional analysis is necessary to establish the relationship between pore type and environmental conditions (e.g., Frenzel et al. 2016). Culturing ostracods under controlled conditions, for example, could provide insights into how variations in salinity affect pore types. Similarly, incorporating internal and external pore types as an additional diagnostic character of valve features allows for the delineation of phylogenetic relationships among species. These pores have been identified as potentially conservative characters, shared even at the species and family levels (Tsukagoshi 1990).

### ﻿Significance of female genital lobes in ostracod taxonomy

The morphology and chaetotaxy of soft body parts is similar among the three *Heterocypris* species, which has already been noted for *H. incongruens* and *H. salina* by Meisch (2000). However, our study reveals morphological differences in the shapes of female genital lobes.

**Figure 16.**
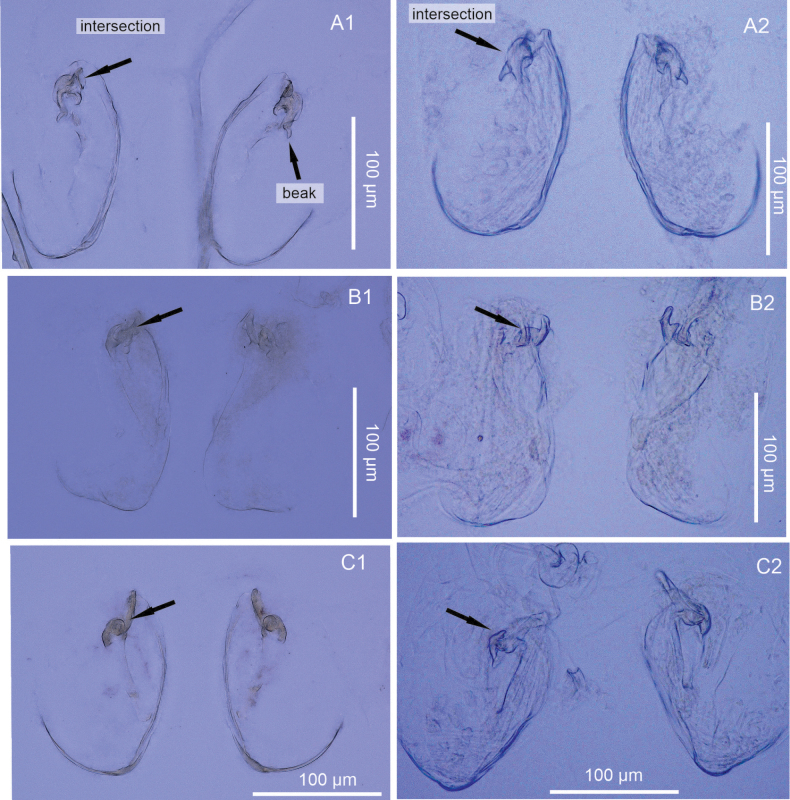
Comparison of female genital lobes in three species of *Heterocypris* Claus, 1892. of *Heterocypris exodonta* Bonilla-Flores & Karanovic, sp. nov. A1. From Nam Co; A2. From Taro Co, Southern Tibetan Plateau. *Heterocypris incongruens* (Ramdohr, 1808); B1. From Mexico City, Mexico; B2. From Taro Co. *Heterocypris salina* (Brady, 1868); C1. From Braunschweig, Germany; C2. From Taro Co. Black arrows indicate the intersection between genital lobes.

Although slight variations were observed in the shape of the intersection of the female genital lobes among specimens of *H. incongruens* from Taro Co, STP, and Mexico City, Mexico, as well as in *H. salina* from Taro Co, and Braunschweig, Germany, these differences can likely be attributed to poor specimen preservation, particularly for those specimens from the STP. Nonetheless, this characteristic shape of the intersection distinguishes *H. exodonta* sp. nov. from *H. incongruens* and *H. salina*, while also highlighting shared traits among populations from different geographic regions (Fig. 16). The most notable difference lies in the sclerotized intersection area, free of signs from post-dissection deformation. However, it is important to note that the preservation of the intersection shape is possible by storage in ethanol. The most distinctive feature of the female genital lobe is the presence of a beak at the intersection in *H. exodonta* sp. nov., which is not observed in *H. incongruens* (Fig. 16B1, B2), while in *H. salina*, the intersection has a hook-like shape (Fig. 16C1, C2).

The female genitalia typically consist of a pair of rounded lobes that house the openings of the vaginas and oviducts. Occasionally, these lobes may have protrusions that males use as anchor points during copulation (Smith and Kamiya 2007). Ostracods are known to engage both sets of sexual organs simultaneously during copulation (Smith 2023). In general, there are few taxonomic studies that comprehensively describe and report on the morphology of the female genital lobes (Purper and Würdig-Maciel 1974; Kong et al. 2014; Mesquita-Joanes et al. 2020). Furthermore, some of these studies have only provided schematic representations of the external view (Broodbakker 1982; Meisch 2000; Karanovic 2012; Matzke-Karasz et al. 2017), where the shape of this structure is not clearly discernible. We propose the use of the female genital lobes to differentiate species with similar morphology, although further analysis and comparisons among different species are necessary to validate their potential as a taxonomic character and their utility in future descriptive studies.

### ﻿Molecular differences

In recent years, the combination of traditional taxonomy, based on morphological characteristics, and DNA barcoding using the CO1 gene for identifying ostracod species has become extremely useful (Nigro et al. 2016). Molecular methods are particularly useful when species share similarities in limb characters and valve morphology (Macario-González et al. 2018). Despite this, there is limited research on molecular analyses in non-marine ostracods in the STP. Echeverría-Galindo et al. (2021) identified eight species based on morphology and molecular data. However, for the species identified as Heterocypris cf. salina, the abbreviation “cf.” and “aff.” was used to indicate uncertainty in its identification due to its resemblance to *H. salina*. However, our study, based on morphological and genetic data, suggests that the sequences originally attributed to Heterocypris cf. salina belong to *H. exodonta* sp. nov., and consequently, the name will be updated in GenBank. Most importantly, the new species forms a distinct branch on the phylogenetic tree and displays high p-distances from all available COI sequences.

Results of our phylogenetic analysis and p-distance calculations suggest that *Heterocypris salina* and *H. incongruens* both represent species complexes (Fig. 14). Our findings complement a recently published study by Kilikowska et al. (2024), which examined 13 populations of *H. salina* from across Europe using 28S rRNA and COI. The authors found that the complex consists of four genetic species, but combining their data with ours is beyond the scope of this paper. While the p-distances observed between Egyptian (Ali et al. 2018) and German populations of *H. salina* are sufficiently high to support their independent species status, the distances between the German *H. salina* and *H. spadix* (described from Japan) are much smaller, which may suggest their synonymy. Unfortunately, Kilikowska et al. (2024) did not incorporate any morphological studies, as is the case with many recent publications on genetic ostracod species (e.g., Bode et al. 2010; Schön et al. 2012, 2017). Cryptic species, if left undescribed, can hinder accurate biodiversity assessments (Platnick 2013), with serious consequences for conservation efforts. Another important result of our analysis is that neither *Heterocypris* nor *Cyprinotus* appears to be monophyletic. This has been suggested previously (Kong et al. 2014; Yoo et al. 2017), and further studies are needed to resolve this issue.

### ﻿Ecology

The three species studied are representatives of shallow and temporary waters, with a capacity for rapid reproduction and colonization of new habitats (Meisch 2000; Mischke 2012). *Heterocypris incongruens* is commonly found in temporary ponds in both the southern and northern parts of the Tibetan Plateau and other parts of the world (e.g., Meisch 2000; Schwalb et al. 2002; Mischke 2012; Akita et al. 2016), where the habitats in the ponds are composed of fine mud and organic matter, providing food (e.g., algae) (Purper and Würdig-Maciel 1974). Subsequently, the phenology of the ostracods responds when these ponds periodically dry, and with the presence of rainwater, populations grow again (Rossi et al. 2011). Rossi et al. (2011) consider *H. incongruens* as an r-strategy species, meaning it is an opportunistic species, colonizing temporary ponds, with a short generation time, reproduction mainly by parthenogenesis, rapid population growth, and even cannibalistic behavior. The latter could influence population density regulation. These ecological strategies result in genetic variability, with a high level of clonal diversity (Rossi and Menozzi 2012).

*Heterocypris exodonta* sp. nov. is found in temporary ponds on the Southern Tibetan Plateau, and it can be considered a freshwater ostracod, also with r-type ecological strategies, similar to *H. incongruens*. During this study, we also observed cannibalistic behavior of adults towards recently molted juveniles. This behavior has multiple hypotheses, including lack of food, competition, and population regulation; however, laboratory studies and observations are required to corroborate these hypotheses (Rossi et al. 2011).

Finally, *Heterocypris salina* can also be found in shallow water bodies (Petkowski et al. 2000), and is considered thermo-euryplastic. It is an indicator of high salinity, with development occurring in summer (Janz et al. 2001). In sediments from Elk Lake, Minnesota, dating to the mid-Holocene, this species was recognized as euryhaline and used as a paleo-indicator of a shallow saline lake (Smith et al. 2002). This is corroborated by the work of Meisch and Broodbakker (1993) on the Canary Islands, where they determined that *H. salina* inhabits water bodies with conductivity ranges between 360–6130 µS/cm.

### ﻿Geographic distribution

*Heterocypris exodonta* sp. nov. has a more restricted geographical distribution, so far, it is known only from the STP. In contrast, *H. incongruens* and *H. salina* have broader distribution ranges spanning Asia, Europe, Africa, and the northern and southern regions of the Americas. However, it is important to note that the cosmopolitan distribution of the latter two species must be confirmed and further examined through molecular analysis to investigate potential cryptic species, as it is confirmed by our molecular study. According to our phylogeny, *H. salina* from Germany and Egypt, as well as *H. incongruens* from Mexico, India, and possibly Turkey, may belong to different species. Our phylogenetic results suggest that *Heterocypris salina* from Germany and Egypt, as well as *H. incongruens* from Mexico, India, and possibly Turkey, may represent distinct species rather than a single cosmopolitan taxon. This pattern aligns with recent findings by Kilikowska et al. (2024), who identified multiple cryptic genetic lineages within *H. salina*, highlighting a higher-than-expected species diversity in morphologically similar populations. These results reinforce the importance of integrative approaches, combining molecular data with morphology and biogeography, to resolve species boundaries in ostracods.

## ﻿Conclusions

The recent discovery and subsequent morphological characterization of *Heterocypris exodonta* sp. nov. from the Southern Tibetan Plateau offers a novel taxonomic perspective within the *Heterocypris* genus. The focus on female genital lobes, particularly the varying shapes of the intersection region of the seminal duct and the oviduct, serves as a crucial diagnostic feature, especially in asexual species and populations where male morphological features are lacking.

This study provides the first detailed description of the internal structure of pore openings in species of the genus *Heterocypris*. The inner morphology was found to differ between species: *H. exodonta* sp. nov. and *H. incongruens* exhibit turbine-shaped internal pores, whereas *H. salina* shows a simple, unstructured aperture. These differences, reported here for the first time within the genus, may serve as valuable diagnostic characters in species delimitation and future systematic studies.

Our molecular analyses, specifically targeting the Cytochrome c Oxidase Subunit 1 gene (CO1), has allowed a comprehensive differentiation of *H. exodonta* sp. nov. from closely related species, *H. incongruens* and *H. salina*. These findings not only reinforce the descriptive aspects of traditional taxonomy but also provide valuable insights into the genetic differences, additionally to observed morphological variations. This meticulous morphological delineation is important in preventing misidentification of *H. exodonta* sp. nov., thus ensuring a clear distinction from other species.

By integrating molecular, morphological, and ecological insights, this study considerably advances our understanding of ostracod biodiversity and evolution. *Heterocypris exodonta* sp. nov. currently has limited records on the STP. Increased sampling efforts in future studies may expand its distribution. In contrast, the widespread distribution of both *H. incongruens* and *H. salina* spans diverse regions, including Asia, Europe, and both northern and southern areas of the Americas, but probably represents species complexes.

## Supplementary Material

XML Treatment for Heterocypris

XML Treatment for Heterocypris exodonta

XML Treatment for Heterocypris incongruens

XML Treatment for Heterocypris salina

## ﻿Additional information

### Use of AI

No use of AI was reported.

### Author ORCIDs

Mauricio Bonilla-Flores https://orcid.org/0000-0002-5593-6522

Ivana Karanovic https://orcid.org/0000-0002-9002-9952

Paula Echeverría-Galindo https://orcid.org/0000-0003-0210-4607

Peter Frenzel https://orcid.org/0000-0002-3821-4632

Liseth Pérez https://orcid.org/0000-0002-5256-3070

Nicole Börner https://orcid.org/0000-0001-8833-6665

Katharina Dulias https://orcid.org/0000-0002-3938-6663

Junbo Wang https://orcid.org/0000-0003-2335-519X

Antje Schwalb https://orcid.org/0000-0002-4628-1958

### Data availability

All of the data that support the findings of this study are available in the main text.
